# Non-canonical imprinting, manifesting as post-fertilization placenta-specific parent-of-origin dependent methylation, is not conserved in humans

**DOI:** 10.1093/hmg/ddaf009

**Published:** 2025-01-17

**Authors:** Dagne Daskeviciute, Louise Chappell-Maor, Becky Sainty, Philippe Arnaud, Isabel Iglesias-Platas, Carlos Simon, Hiroaki Okae, Takahiro Arima, Rita Vassena, Jon Lartey, David Monk

**Affiliations:** Biomedical Research Centre, School of Biological Sciences, University of East Anglia, Norwich Research Park, Earlham Road, Norwich NR4 6PN, United Kingdom; Biomedical Research Centre, School of Biological Sciences, University of East Anglia, Norwich Research Park, Earlham Road, Norwich NR4 6PN, United Kingdom; Biomedical Research Centre, School of Biological Sciences, University of East Anglia, Norwich Research Park, Earlham Road, Norwich NR4 6PN, United Kingdom; Université Clermont Auvergne, CNRS, Inserm, GReD, 49 bd François Mitterrand, Clermont-Ferrand 63001, France; Institut de Recerca, Sant Joan de Déu, C. de Sta. Rosa, 39, Barcelona 08950, Spain; Neonatal Research, Norwich and Norwich University Hospital NHS Foundation Trust, Colney Ln, Norwich NR4 7UY, United Kingdom; Carlos Simon Foundation, Rda. de Narcís Monturiol, 11, Bloque C, 46980 Paterna, Valencia, Spain; Department of Obstetrics and Gynecology, Valencia University and INCLIVA, Av. Blasco Ibáñez 15, Valencia 46012, Spain; Department of Trophoblast Research, Institute of Molecular Embryology and Genetics, Kumamoto University, Kumamoto 860-0811, Japan; Department of Informative Genetics, Environment and Genome Research Center, Tohoku University Graduate School of Medicine, Sendai 980-8575, Japan; Fecundis, C/Baldoro i Reixac 10-12, Barcelona 08028, Spain; Department of Obstetrics and Gynaecology, Norwich and Norwich University Hospital NHS Foundation Trust, Colney Ln, Norwich NR4 7UY, United Kingdom; Biomedical Research Centre, School of Biological Sciences, University of East Anglia, Norwich Research Park, Earlham Road, Norwich NR4 6PN, United Kingdom; Bellvitge Biomedical Research Institute, Avinguda de la Granvia de l’Hospitalet 199, L’Hospitalet de Llobregat, Barcelona 08908, Spain

**Keywords:** Imprinting, DNA methylation, placenta, pre-implantation embryos

## Abstract

Genomic imprinting is the parent-of-origin dependent monoallelic expression of genes often associated with regions of germline-derived DNA methylation that are maintained as differentially methylated regions (gDMRs) in somatic tissues. This form of epigenetic regulation is highly conserved in mammals and is thought to have co-evolved with placentation. Tissue-specific gDMRs have been identified in human placenta, suggesting that species-specific imprinting dependent on unorthodox epigenetic establishment or maintenance may be more widespread than previously anticipated. Non-canonical imprinting, reliant on differential allelic H3K27me3 enrichment, has been reported in mouse and rat pre-implantation embryos, often overlapping long terminal repeat (LTR)-derived promoters. These non-canonical imprints lose parental allele-specific H3K27me3 specificity, subsequently gaining DNA methylation on the same allele in extra-embryonic tissues resulting in placenta-specific, somatically acquired maternal DMRs. To determine if similar non-canonical imprinting is present in the human placenta, we interrogated allelic DNA methylation for a selected number of loci, including (i) the human orthologues of non-canonical imprinted regions in mouse and rat, (ii) promoters of human LTR-derived transcripts, and (iii) CpG islands with intermediate placenta-specific methylation that are unmethylated in gametes and pre-implantation embryos. We failed to identify any non-canonical imprints in the human placenta whole villi samples. Furthermore, the assayed genes were shown to be biallelically expressed in human pre-implantation embryos, indicating they are not imprinted at earlier time points. Together, our work reiterates the continued evolution of placenta-specific imprinting in mammals, which we suggest is linked to epigenetic differences during the maternal-to-embryo transition and species-specific integration of retrotransposable elements.

## Introduction

Elegant pronuclear transplantation experiments revealed that genetic contributions from each gamete are essential for mammalian development since uniparental mice die early in gestation [1, 2]. It was subsequently postulated that the parental genomes were differentially marked during gametogenesis which would result in “genomic imprinting” after fertilization, the monoallelic parental allele-specific expression observed in monotremes, mammals and flowering plants [3]. It has been hypothesized that imprinting arose in placental mammals to regulate maternal resources during gestation [4], or that maternal silencing would impede parthenogenetic oocyte activation [5].

The vast majority of mammalian imprints are established early in development as a consequence of germline-derived DNA methylation, which is deposited in respective gametes resulting in life-long allele-specific methylation [6, 7]. Curiously, there is a bias for germline differentially methylated regions (gDMRs) to be established in oocytes, which reflects the requirement for active transcription during the process. Imprinted loci regulated by gDMRs is referred to as “canonical” imprinting, which in mouse requires the combined action of DNA methyltransferase 3a (DNMT3A), DNMT3B and its catalytic inactive partner DNMT3L are recruited to target loci by an underlying histone modification landscape [8, 9]. DNA methylation in oocytes is higher at transcriptionally active regions, which are associated with histone 3 lysine thirty-six trimethylation (H3K36me3) [10]. This modification is deposited by SETD2, which is recognised by the PWWP domain of DNMT3A [11]. Additional biochemical studies have shown that the ADD domain of DNMT3L interacts with histone H3, but only when this lysine 4 is unmethylated. H3K4 di- and trimethylation are removed by the germline-specific H3K4 demethylase KDM1B/AOF1 highlighting further interplay between histone modifications and the DNA methylation machinery in coordinating the establishment of gDMRs [12–14]. Whilst these elegant processes are essential for establishing gDMRs in mice, the absence of DNMT3L expression in human GV-metaphase II oocytes suggests that *de novo* methylation occurs independently in the human female germline [15].

The vast majority of ubiquitous imprinted gDMRs identified in mice are conserved in humans. Of the 21 known gDMRs in the mouse genome, all are observed in humans except *Rasgrf1, Impact* and *Zrsr1/U2af1-rs1*. Of the 36 ubiquitous gDMRs identified in humans [16], only 19 are evolutionarily conserved in mice, suggesting that the number of imprinted genes is under evolutionary expansion during the last ~90 million years. This is supported by the recent description of placenta-specific imprinting, which is largely exclusive to humans [17]. To date, ~150 oocyte-derived gDMRs have been confirmed to maintain maternal methylation in the placenta tissues only [18–22]. In 2017, the list of imprinted loci was further expanded by the description of non-canonical imprinting in mice [22]. These genes do not require gDMRs as DNMT-deficiency in oocytes did not affect imprinted expression in the placenta [23]. Instead imprinting relied on maternally-derived H3K27me3, a repressive histone modification for transient imprinting in pre-implantation embryos. H3K27me3 enrichment colocalized with H2AK119ub in oocytes, catalysed by the polycomb repressive complexes PRC1 and PRC2, resulting in maternal allele-specific silencing for *Sfmbt2*, *Phf17/Jade1*, *Gab1*, *Sall1*, *Platr20*, *Smoc1*, *Slc38a4* and *Xist* [22, 24]. Maternal H3K27me3 and H2AK119ub are not maintained beyond pre-implantation development [25, 26], with paternal expression preserved in the placenta by the establishment of secondary DMRs (sDMRs), requiring DNMT3A/3B. Furthermore, endogenous retrovirus-K (ERVK) long terminal repeats (LTRs) are also involved in non-canonical imprinting [27]. Several of these endogenous retroviral elements act as alternative promoters for chimeric imprinted transcripts that exhibit paternal allele-specific accumulation of H3K4me3 with the maternal alleles enriched for H3K27me3. These LTRs also transition to sDMRs in extra-embryonic tissues, maintaining imprinting in the placenta throughout gestation. Interestingly, a similar mechanism leading to non-canonical imprinting was recently described in rats [28]. In addition to confirming imprinting for *Sfmtb2, Gab1* and *Sall1*, Albert and colleagues also discovered eight novel non-canonical imprinted genes unique to rat. This suggests that imprinting is continuing to evolve in extra-embryonic lineages, since the establishment of rat-specific imprinted genes must have occurred within the last 13 million years since the divergence from mice.

While loci subject to canonical imprinting, and their underlying molecular mechanisms, show evolutionary conservation between mice and humans, the same cannot be said for placenta-specific gDMRs, which are largely absent in other mammals [7, 18]. The presence of non-canonical imprinting in the human genome is currently unreported. Here we describe the characterisation of human orthologues of non-canonical imprinted genes in mouse and rat, as well as a systematic screen for human non-canonical imprints that manifest as placenta-specific sDMRs. Our study reveals that non-canonical sDMRs are not present in the human placenta, suggesting that imprinting in humans is dependent on the establishment of gDMRs.

## Results

### Orthologues of mouse non-canonical imprinted genes are not imprinted in humans

**Figure 1:**
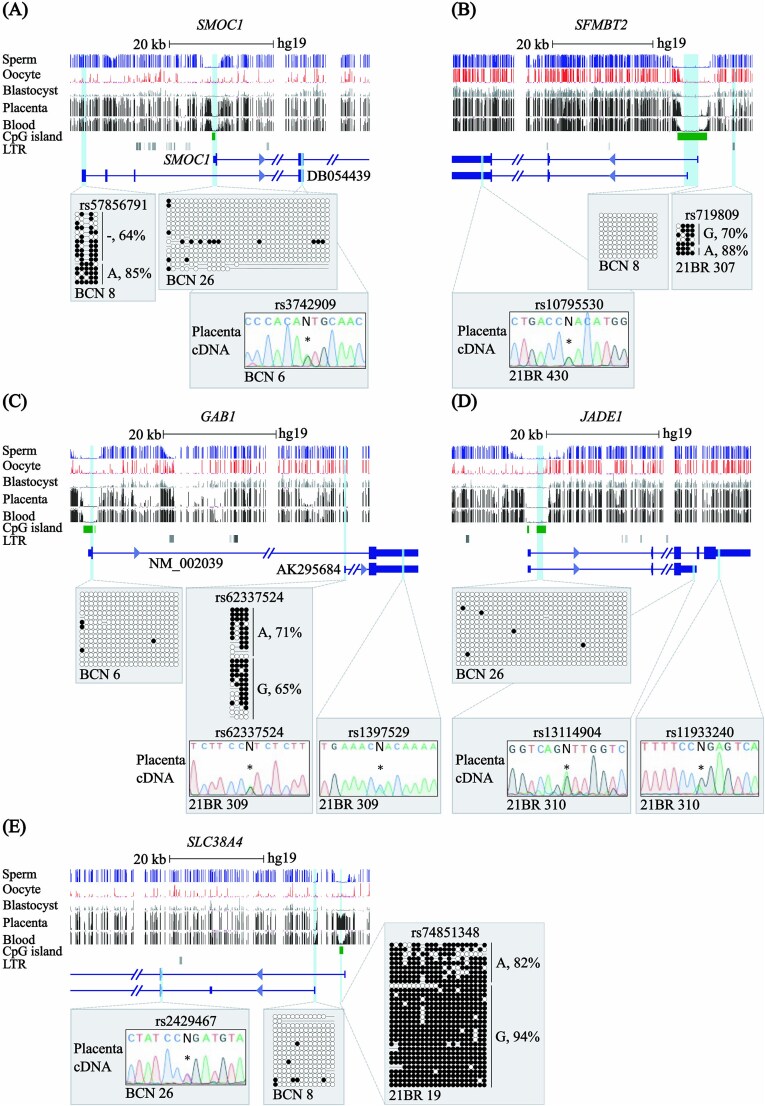
Characterisation of DNA methylation and allelic expression for the human orthologues of non-canonical imprinted mouse genes. (A) Genomic maps of the human intervals containing (A) *SMOC1,* (B) *SFMBT2*, (C) *GAB1*, (D) *JADE1* and (E) *SLC38A4* genes. CpG islands and the exons of each transcript are shown. The locations of the mouse placenta-specific sDMRs are indicated. For each gene, the DNA methylation profiles observed in methyl-seq datasets are shown. The vertical lines in the methyl-seq tracks represent the mean methylation values for individual CpG dinucleotides. Promoter methylation was confirmed using bisulphite PCR and sub-cloning in placenta-derived DNA. Each circle represents a single CpG on a DNA strand. Allelic methylation shown as %. (•) Methylated cytosine, (o) unmethylated cytosine. Each row corresponds to an individual cloned sequence with the parent-of-origin indicated by the genotype of SNPs if heterozygous. Sequence traces of RT-PCR products incorporating SNPs for *SFMBT2* (rs10795530*), JADE1* (rs13114904 & rs11933240) and *SMOC1* (rs3742909), G*AB1* (rs62337524 & rs1397529) and *SLC38A4* (rs2429467) genes are shown.

To determine whether any of the non-canonical imprinted genes identified in the mouse placenta are imprinted in humans, we screened our genome-wide methyl-seq datasets [7] and assessed allelic DNA methylation using bisulphite PCR and expression using targeted RT-PCR approaches in human placenta biopsies. Analysis of four orthologous genes that exhibit non-canonical imprinted expression in mouse extra-embryonic lineages revealed a lack of allelic DNA methylation at the equivalent sDMR locations observed in mice. The *SMOC1* gene possesses an upstream transcription start site (TSS) (DB054439) that originates from a LINE-L2b element that is fully methylated in the placenta (Fig. 1A). When informative single nucleotide polymorphisms (SNPs) were identified, biallelic expression was observed for *SMOC1, SFMBT2*, *JADE1* (also known as *PHF17*) and *GAB1* (Table 1) (Fig. 1; Supplementary Material, Table S1). Biallelic expression of *SFMBT2* confirms previous reports that this gene is not imprinted in humans, an observation that correlates with the absence of a large block of micro-RNAs in intron 10 of the gene [29]. Interestingly there is an ERVL-MaLR retrotransposon in humans approximately 5 kb upstream of the *SFMBT2* TSS, in a similar position as the RLTR11B-ERVK LTR identified as an alternative promoter for mouse *Sfmtb2* [27, 28], however, this interval is methylated on both parental alleles in human placenta (Fig. 1B). In the case of *GAB1*, the human orthologous interval possesses an alternative transcript originating from a comparable intergenic location (AK295684) as the imprinted isoform in mouse [27]. Despite this similar genomic organisation, isoform-specific expression and promoter methylation analysis revealed a robust lack of imprinting in term placenta for both *GAB1* isoforms, consistent with the absence of the ERVK LTR in the human genome (Fig. 1C). The human orthologue of a fifth non-canonical imprinted gene that is regulated by both maternal H3K27me3 and DNA methylation in mouse placenta, *Slc38a4* [22, 27, 30], was also found to lack a DMR overlapping the equivalent promoter interval and was biallelically expressed in numerous placenta samples (Fig. 1E). Two other non-canonical imprinted genes in mouse, *Gm32885* and *Platr20* [30], could not be assessed in humans due to the absence of annotated orthologues.

**Table 1:** List of mouse non-canonical imprinted genes and their status in humans as revealed in this study.

**Gene**	**Mouse**		**Rat**		**Human**	
	**Allelic expression**	**Allelic methylation**	**Allelic expression**	**Allelic methylation**	**Allelic expression**	**Allelic methylation**
*Sfmbt2* Ref [22, 24, 29]	Paternal	mat sDMR	Paternal	mat sDMR	Biallelic	ERVL promoter methylated; major promoter unmethylated
*Gab1* Ref [22, 23, 27]	Paternal	mat sDMR	Paternal	mat sDMR	Biallelic	Unmethylated
*Slc38a4* Ref [22, 24, 27]	Paternal	mat gDMR	Paternal	mat sDMR	Biallelic	Unmethylated
*Sall1* Ref [22, 24, 28]	Paternal	mat sDMR	Paternal	mat sDMR	Biallelic	Mosaic
*Smoc1* Ref [22, 24]	Paternal	mat sDMR	nd	nd	Biallelic	LINE promoter methylated; major promoter unmethylated
*Jade1* Ref [22, 24, 27]	Paternal	mat sDMR	nd	nd	Biallelic	Unmethylated
*Xist* Ref [22, 28]	Paternal	Mat sDMR	Paternal	Mat sDMR	Biallelic	Random allelic

### Orthologues of rat non-canonical imprinted genes are not imprinted in humans

**Figure 2:**
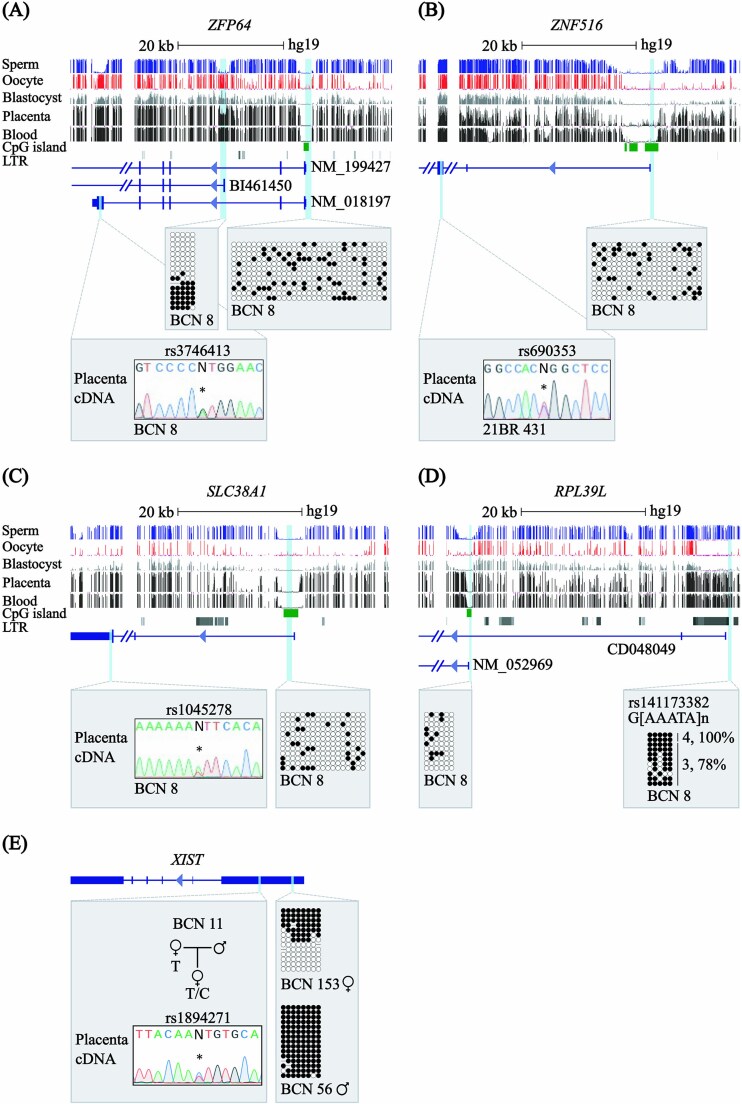
Study of DNA methylation and allelic expression for the human orthologues of non-canonical imprinted rat genes. Genomic maps of the human intervals containing (A) *ZFP64,* (B) *ZNF516* and (C) *SLC38A1* genes. CpG islands and the exons of each transcript are shown. The locations of the rat placenta-specific sDMRs are indicated. For each gene, the DNA methylation profiles observed in methyl-seq datasets are shown. The vertical lines in the methyl-seq tracks represent the mean methylation values for individual CpG dinucleotides. Promoter methylation was confirmed using bisulphite PCR and sub-cloning in placenta-derived DNA. Each circle represents a single CpG on a DNA strand. Allelic methylation shown as %. (•) Methylated cytosine, (o) unmethylated cytosine. Each row corresponds to an individual cloned sequence with the parent-of-origin indicated by the genotype of SNPs if heterozygous. Sequence traces of RT-PCR products incorporating SNPs for *ZFP64* (rs3746413), *ZNF516* (rs690353) and *SLC38A1* (rs1045278) genes are also shown. (D) Genomic map for the human *RPL39L* loci on chromosome 3, showing the representative methyl-seq profiles for the LTR-derived chimeric transcript. The DNA methylation profiles at the retrotransponon-associated promoter and main TSS were confirmed in placenta-derived DNA by bisulphite PCR. (E) Gene structure of *XIST*, showing biallelic expression (rs1894271) in female placenta tissue. The DNA methylation profiles show differing signatures for male and female-derived placenta DNA samples.

In addition to the above characterised non-canonical imprinted genes, the rat also possesses several imprinted genes that are marked by allelic H3K27me3 independent of germline DNA methylation which ultimately switch to placental sDMRs (Table 2) [28]. The promoter intervals of the human orthologues of *Zfp64*, *Zfp516* and *Slc38a1* genes were all unmethylated in the human placenta (Fig. 2A–C; Supplementary Material, Table S1). Interrogation of our methyl-seq dataset revealed a maternally-methylated DMR within intron 2 of *ZFP64*, overlapping Alu repeats and the first exon of BI461450, that inherits methylation from the oocyte (Fig. 2A). This region is 15.6 kb from the orthologous non-canonical sDMR in rats. Informative exonic SNPs were identified, allowing for allelic discrimination of expression in *ZFP64*, as well as *ZFP516* and *SLC38A1*, all of which were biallelically expressed in term placenta samples (Fig. 2). No expression from BI461450 was detected in placenta samples. A forth rat non-canonical imprinted gene, *Rpl39l*, also has an orthologue in humans. The gene expresses two isoforms, the longest (CD048049) originating from an ERV1 LTR element which we show is fully methylated in the human placenta (Fig. 2D). Unfortunately, we could not determine allelic expression for this gene due to the lack of heterozygous for exonic SNPs in our sample set.

**Table 2:** List of rat non-canonical imprinted genes and their status in humans as revealed in this study.

**Gene**	**Rat**		**Human**	
	**Allelic expression**	**Allelic methylation**	**Allelic expression**	**Allelic methylation**
*Zfp64* Ref [28]	Paternal	mat sDMR	Biallelic	Unmethylated
*Zfp516* Ref [28]	Paternal	mat sDMR	Biallelic	Unmethylated
*Rpl39l* Ref [28]	Paternal	mat sDMR	N/A	LTR promoter methylated; major promoter unmethylated
*Slc38a1* Ref [28]	Paternal	mat sDMR	Biallelic	Unmethylated

As further evidence of a lack of imprinting of the human orthologues for the mouse and rat non-canonical imprinted genes in human placenta samples, we interrogated their DNA methylation and expression in trophoblast stem cells from biparental (CT^30^) and androgenic moles (CT^mole#1^) [31, 32]. The expression of two known paternally expressed genes were used as controls. Both *PEG10* and *DNMT1* were expressed 2-fold in CT^mole#1^ compared to CT^30^, consistent with two active paternally-derived chromosomes. Only a few genes were expressed in these lines, with *GAB1, SFMBT2, SLC38A4, SLC38A1* and *ZFP64* being expressed at equivalent levels from unmethylated promoters (Supplementary Material, Fig. S1; Supplementary Material, Table S2), supporting that they are not imprinted in human trophoblasts. Furthermore, we found no evidence of allelic methylation in immune-enriched placenta cell-types (Supplementary Material, Table S2).

### Non-imprinted expression of XIST in human placenta

To achieve dosage compensation in mammals, one of the two X chromosomes in females is transcriptionally silenced in the developing embryo. In the case of X-inactivation in mouse, DNA methylation is acquired at the last stage of this epigenetic cascade, which begins with the expression of the non-coding *Xist*, ultimately coating the designated X-chromosome for inactivation, which triggers heterochromatization [33]. In placental cells of both mice and rats, the paternally inherited X chromosome is preferentially inactivated, likely due to H3K27me3-mediated non-canonical imprinting of *Xist* [22, 28]. Using allelic RT-PCR, we observe biallelic *XIST* expression consistent with random monoallelic expression and non-imprinted X-chromosome inactivation. Furthermore, the interval overlapping *XIST*-P2 promoter has a signature consistent with being allelically methylated in female-derived placenta samples and hypermethylation in male-derived placenta samples concordant with a lack of expression in males (Fig. 2E).

### The *FAM101A* gene is not imprinted in the human placenta

**Figure 3:**
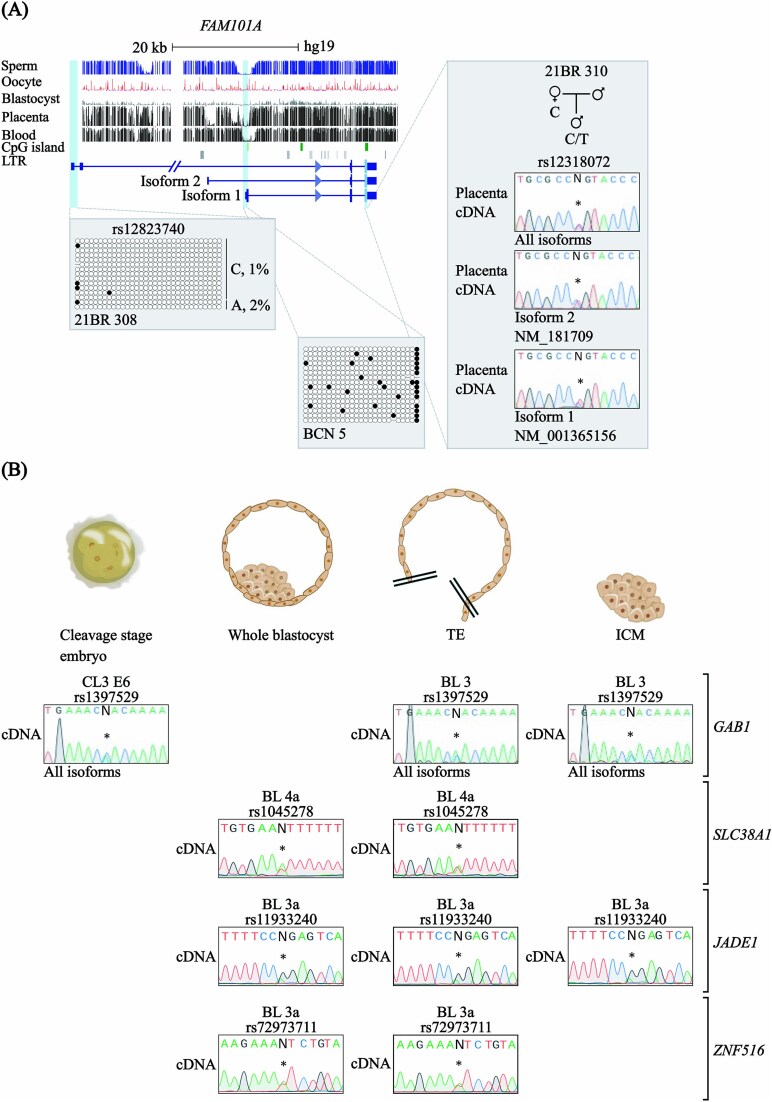
Temporal expression on non-canonical imprinted candidates in human pre-implantation embryos. (A) Schematic map of the human *FAM101A* locus showing promoter methylation profiles and allelic expression patterns in term placenta. DNA methylation was confirmed using bisulphite PCR and sub-cloning in placenta-derived DNA. Each circle represents a single CpG on a DNA strand. Allelic methylation shown as %. (•) Methylated cytosine, (o) unmethylated cytosine. Each row corresponds to an individual cloned sequence with the parent-of-origin indicated by the genotype of the rs12823740 SNP. (B) Allelic expression patterns in human pre-implantation embryos at cleavage day 3 and blastocysts (complete and surgically separated into ICM and TE). Sequence traces of RT-PCR products incorporating SNPs for *GAB1* (rs1397529 and rs28924077), *SLC38A1* (rs1045278 and rs3498), *JADE1* (rs11933240) and *ZNF516* (rs72973711) genes are also shown.

Recent studies have attempted to determine whether asymmetric H3K27me3 distribution in human pre-implantation embryos correlates with allelic gene expression [34]. *FAM101A* was reported to exhibit maternal-biased H3K27me3 and paternal-biased expression in two embryos from one donating couple, suggesting that non-canonical imprinting may be present specifically at early developmental stages, but to our knowledge the imprinting status of this gene has not been assessed in the placenta. We subsequently performed promoter bisulphite PCR and isoform-specific RT-PCR for *FAM101A* and observed robust biallelic expression from unmethylated promoters in placenta samples (Fig. 3A; Supplementary Material, Table S3).

### Non-canonical imprinted gene orthologues are not imprinted in human pre-implantation embryos

Since the monoallelic expression of non-canonical imprinted genes first occurs via allelic H3K27me3 during the zygotic-to-maternal transition in mouse and rat embryos, we wanted to know if the human orthologues of these genes show temporal imprinting during pre-implantation developmental stages. RT-PCR across highly polymorphic SNPs on post-amplified individual embryo RNAs revealed biallelic expression for *GAB1* in cleavage stage embryos (4–16 cell stage) and blastocysts (day 6), the latter surgically separated into inner cell mass (ICM) and trophectoderm (TE) (Fig. 3B; Supplementary Material, Table S3). Biallelic expression was also observed for *SLC38A1, JADE1 and ZNF516* in blastocysts.

### Human LTR-derived chimeric transcripts are not associated with sDMRs in the placenta

**Figure 4:**
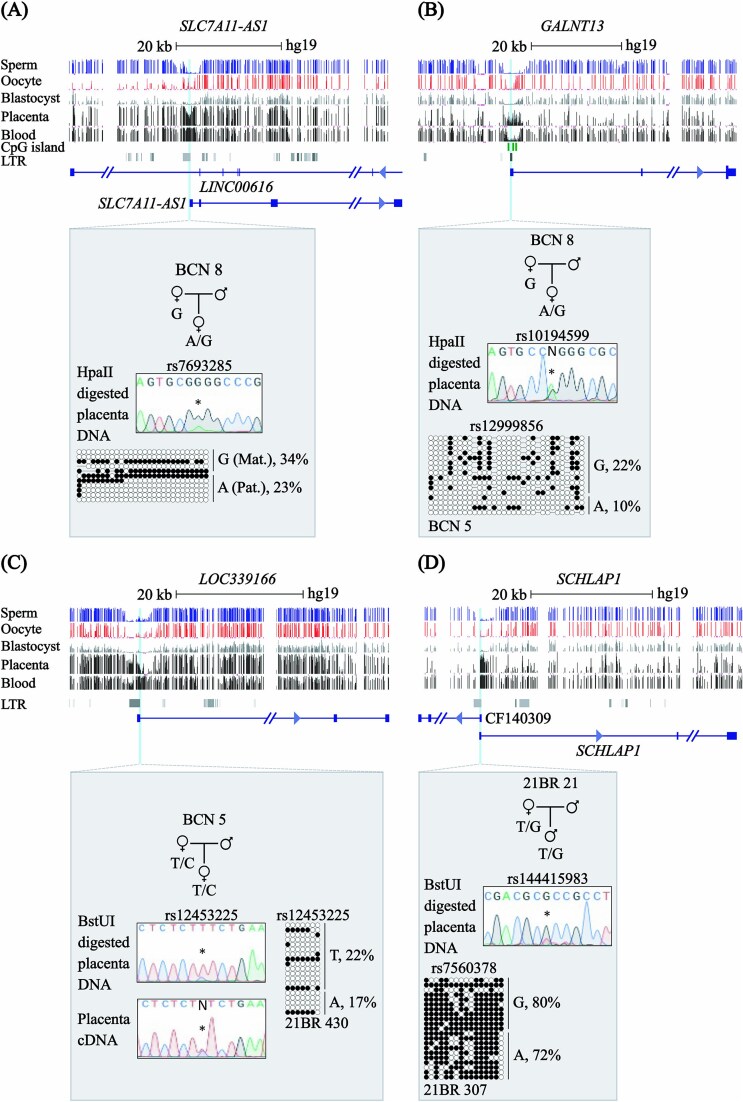
Allele-specific DNA methylation profiling of human LTR-associated promoters with partially methylated profiles in placenta. Maps of the genomic intervals associated with autosomal ERV-chimeric transcripts with partial methylation in placenta methyl-seq. Exonic sequences of each transcript are shown. LTR locations represent those identified by RepeatMasker. The DNA methylation profiles for (A) *SLC7A11-AS1*, (B) *GALNT13,* (C) *LOC339166* and (D) *SCHLAP1*, as observed in methyl-seq datasets are shown. The vertical lines in the methyl-seq tracks represent the mean methylation values for individual CpG dinucleotides. For each gene, methylation-sensitive genotyping and bisulphite PCR and sub-cloning was used to confirm the DNA methylation profiles in placenta-derived DNA. Each circle represents a single CpG on a DNA strand. Allelic methylation shown as %. (•) Methylated cytosine, (o) unmethylated cytosine. Each row corresponds to an individual cloned sequence with the parent-of-origin indicated by the genotype of heterozygous SNPs.

To refine our approach to identify human non-canonical sDMRs in the placenta, we took advantage of the fact that many of these genes in mouse and rat are derived from alternative promoters embedded within solo-LTR of the ERVK family of retrotransposons [26, 27] and that several human placenta-specific gDMRs are associated with species-specific ERV elements [27, 35, 36]. We subsequently interrogated our methyl-seq datasets for 1165 reported autosomal ERV-chimeric transcripts [35, 37] for a methylation profile consistent with being a placenta-specific sDMR. Of these, *SLC7A11-AS1*, *GALNT13, LOC339166* and *SCHLAP1* were partially methylated in placenta but unmethylated in sperm, oocytes and pre-implantation embryos (Fig. 4A–D; Supplementary Material, Table S4). To determine if methylation was restricted to one parental allele in the placenta, we employed methylation-sensitive genotyping assays to those intervals containing highly informative polymorphisms. This method involves allele-calling on genomic DNA before and after digestion with the methylation-sensitive endonucleases, HpaII or BstUI. Allelic methylation is confirmed when a heterozygous genomic DNA sample is reduced to homozygosity following digestion with the remaining allele representing the methylated chromosome for which the genotype can be phased with those obtained from parental samples (Supplementary Material, Fig. S2). We have previously used this method to successfully identify imprinted DMRs [7, 18, 38] as it can readily distinguish between imprinted, random monoallelic and mosaic methylation. All samples showed evidence of biallelic methylation which was confirmed using bisulphite treatment, followed by PCR amplification, cloning of PCR products and sequencing for all four loci. Taken together, these results indicate that differential epigenetic marking of parental alleles at LTR retrotransposons is not a common mechanism resulting in non-canonical imprinting in the human placenta.

### Systematic screen for placenta-specific sDMRs fails to identify non-canonical imprints

**Figure 5:**
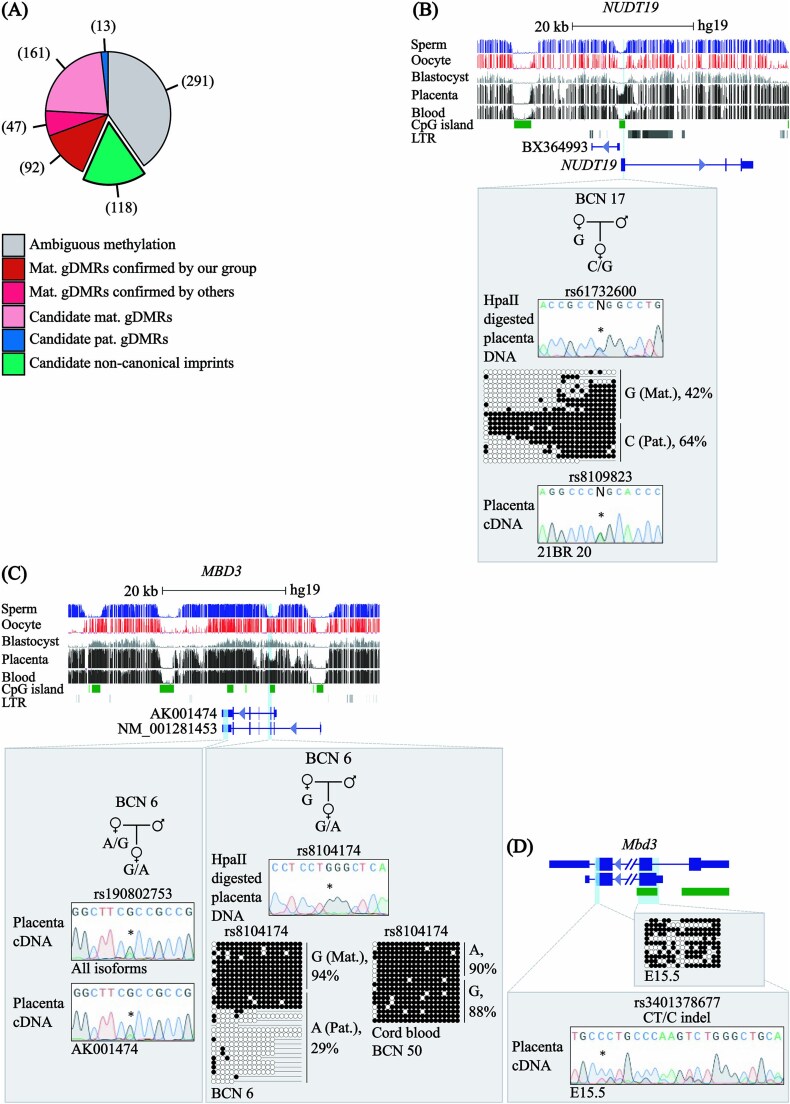
Systematic screen for placenta-specific sDMRs. (A) Pie chart showing the breakdown of germline-derived methylation for the 722 partially methylated placenta domains identified by methyl-seq. (B) Map of the genomic interval associated with *NUDT19* in placenta methyl-seq, with informative methylation-sensitive genotyping (rs61732600) and bisulphite PCR and sub-cloning confirming a lack of parent-specific methylation. Biallelic expression was observed for rs8109823. (C) Confirmation of maternal methylation at the *MBD3* gDMR and the subsequent developmental hypermethylated switch in somatic tissues. Bisulphite PCRs on placenta and cord blood derived-DNA samples were used for confirmation. Each circle represents a single CpG dinucleotide on a DNA strand. Allelic methylation shown as %. (•) Methylated cytosine, (o) unmethylated cytosine. Each row corresponds to an individual cloned sequence. Sequence traces of RT-PCR products for *the MBD3* isoforms are also shown. (D) Allelic methylation and expression analysis of the murine *Mbd3* ortholog in placenta of C57BL6 with JF1 intersubspecific mouse cross.

Whilst direct cross-species characterisation of allelic expression and DNA methylation failed to identify non-canonical imprinting for rat and mouse orthologous genes in human placenta samples, it is possible that the human genome contains unique, non-conserved, non-canonical imprinted genes. To facilitate the screening for non-canonical imprints in the human placenta, we employed an initial screening approach to identify partially methylated regions present solely in our placenta methyl-seq dataset using a sliding within approach (0.25 < mean of 25 CpGs ± 2SD < 0.75). These criteria would readily identify all imprinted gDMRs (ubiquitous gDMRs average 105 ± 73 CpGs; placenta-specific gDMRs 114 ± 56 CpGs), as well as full-length ERVK (125 CpGs), but not solo-LTRs (18 CpGs), and 10/11 known mouse and rat non-canonical DMRs (average 78 ± 46 CpGs, based on mouse placenta methyl-seq [39].

(Supplementary Material, Table S5). This revealed 722 partially methylated intervals with ~50% methylation in the placenta, of which 118 possessed no evidence of germline or allelic methylation in blastocysts or somatic tissues, 94 of which were associated with genes (Supplementary Material, Table S6) (Fig. 5A; Supplementary Material, Table S7). Despite screening 17 loci fulfilling these criteria that possessed informative SNPs, all were randomly methylated on both parental alleles (Supplementary Material, Fig. S3). As highlighted by *NUDT19*, methylation was observed on both alleles following methylation-sensitive genotyping, with mosaic placental methylation confirmed using bisulphite PCR sub-cloning associated with biallelic expression (Fig. 5B).

Recently, Hanna and Kelsey identified 65 regions that were considered candidates for human non-canonical imprints [40]. These loci were unmethylated in oocytes and enriched for H3K27me3, as well as possessing a partially methylated profile in the placenta, although the allelic origin of the methylation was not determined. Direct interrogation of our methyl-seq datasets revealed that only one of the 65 regions*, C5ORF38,* was partially methylated in our placenta methyl-seq dataset and unmethylated in both gametes and pre-implantation embryos. Methylation-sensitive genotyping revealed robust biallelic methylation discounting *C5ORF38* as a non-canonical sDMRs (Supplementary Material, Table S6).

### Methylation-sensitive genotyping reveals eight novel oocyte-derived gDMRs

The majority of the 722 partially methylated intervals with ~50% methylation in the placenta are associated with oocyte-derived germline methylation, of which 139 have already been confirmed using alternative allelic strategies (Fig. 5A) [7, 18–21, 38]. To further characterise the remaining 161 candidate maternal gDMRs, we screened for loci containing highly informative SNPs within promoter CpG islands. We performed methylation-sensitive genotyping for eight candidate loci, including *DYRK1B*, *LRRC8D*, *WNT7B*, *CLDN23*, *WNT7B*, *PRKAG2, STARD13* and *MBD3*. All eight regions were maternally methylated in term placenta and with the exception of *MBD3*, were unmethylated in somatic tissues (Supplementary Material, Fig. S4; Fig. 5C; Supplementary Material, Table S7). The maternally methylated CpG island associated with *MBD3* that overlaps intron 2 of the full-length *MBD3* transcript (NM_001281453), which become fully methylated in all somatic tissues (Fig. 5C). This scenario is shared with only five placenta-specific gDMRs to date: *C19MC*, *GRID2*, *TMEM247*, *GPR1-AS1* and *ZFAT* [18, 41–43]. To determine if this interval was the promoter of a novel transcript in the placenta, we performed 5’RACE, which revealed a unique TSS mapping within the gDMR with high sequence identity with the Expressed Sequence Tag AK001474. Subsequent allele-specific RT-PCR revealed preferential allelic expression for this novel transcript in one placenta sample which was unfortunately not informative as the accompanying maternal DNA sample was also heterozygous (Fig. 5C). In line with previous observations for placenta-specific gDMRs, the orthologous region in mouse placenta samples from intersubspecific mouse crosses (C57BL/6 x JF1) was not allelically methylated and was biallelically expressed at embryonic day 15.5 (Fig. 5D).

## Discussion

For more than 30 years, there have been community-wide endeavours to systematically characterise imprinting in different species, with much effort given to comparing mouse-human orthologues. This has revealed that the conservation of imprinting status is largely dependent on the molecular mechanisms leading to monoallelic expression. For example, approximately half of the repertoire of ubiquitously imprinted transcripts in the mouse show conserved imprinting pattern in humans [28]. However, many mouse imprinted genes are not conserved in humans. This was first systematically reported for placenta-specific transcripts with monoallelic expression in mice that depend upon allelic H3K9me3 and H3K27me3 within the *Kcnq1* and *Igf2r* domains [44]. Subsequent studies have shown that the vast majority of human imprinted transcripts originating from within oocyte-derived placenta-specific gDMRs are also not conserved in other mammalian species, including mouse, cow, dog and macaque [18].

To continue these efforts, we have taken advantage of our extensive collection of placental samples and developmental series of methyl-seq datasets that include human gametes, pre-implantation embryos and placenta to screen for non-canonical imprinting in humans. Uniquely, non-canonical imprinted genes in mouse are first established via an allelic imbalance of H3K27me3, which is subsequently replaced by sDMRs selectively in extra-embryonic tissues [22], an epigenetic signature we could exploit in our datasets. To our surprise, we identified numerous promoter intervals that possessed placenta-specific partial methylation consistent with being a sDMR, but upon allelic characterisation did not manifest as allelically enriched. Reassuringly, our approach utilizing methylation-sensitive genotyping did identify a further eight placenta-specific gDMRs originating from the oocytes, confirming that our approach could readily discriminate allelic DNA methylation if present. To ensure our screen for sDMRs associated with human non-canonical imprinting was exhaustive, we further profiled the DNA methylation of human LTR-fusion transcripts since some non-canonical imprinted genes in mice and rats are associated with these repetitive genomic elements. The orthologues of these genes did not possess a maternal sDMR and were robustly expressed from both parental alleles in the human placenta, which was not surprising given that the retrotransposons implicated in their establishment are rodent-specific. This is highlighted by *Gab1* sDMR which overlaps with an alternative promoter interval originating within an ERVK LTR that drives paternal-specific expression which is absent in the human genome. However, despite the retrotransposon not being conserved, it remained a possibility that the mechanism could still give rise to human-specific non-canonical imprints as LTR-initiated transcription is associated with at least 10% of human placenta-specific gDMRs [27]. We screened 1165 LTR-derived fusion transcripts reported in the human genome, of which four looked like promising candidates upon methyl-seq interrogation. Unfortunately, all candidates were mosaically methylated on both alleles, rather than being a maternally-methylated sDMRs.

In 2019 Zhang and colleagues identified multiple regions with preferential maternal enrichment of H3K27m3 independent of DNA methylation in human pre-implantation embryos for which paternal allele expression bias was suggested from RNA-seq analysis [34]. One gene, *FAM101A* was reported as monoallelic in two embryos, although monoallelic expression at later developmental stages, or in the placenta was not described. We interrogated the genomic interval encompassing the *FAM101A* gene and did not observe evidence of a placenta-specific sDMR. Unfortunately, none of our pre-implantation embryos were heterozygous for *FAM101A*, although we did observe widespread biallelic expression for *GAB1*, *SLC38A1, JADE1 and ZNF516*, confirming that human orthologues are not imprinted in a temporal fashion.

Although post-fertilization events are largely conserved between mammals, there are species-specific differences between human and mice that may influence the establishment of non-canonical imprinting. Firstly, mice having multiple gestations compared to human singleton deliveries. In addition, there are also notable anatomical distinctions. Both mouse and human have haemochorial placentae. In the mouse labyrinth, three layers of trophoblast separate maternal and fetal blood, while in the term human placenta, there is only one functional layer of trophoblast separating maternal and fetal blood [45]. At the cellular level, both possess differentiated trophoblast cells; syncytiotrophoblast and anchoring trophoblasts that attach the placenta to the uterine wall. In mouse these are the giant cells or glycogen trophoblasts, whereas the human equivalent are called extravillous trophoblasts. At the epigenetic level, several histone modifications show non-canonical distributions and functions in mouse oocytes and early pre-implantation embryos, including H3K4me3 and H3K27me3, which display “broad” domains rather than distinct peaks of enrichment observed at the equivalent human stages of development [46, 47]. However, the role of the “broad” peaks in non-canonical imprinting is questionable since *Gab1* and *Slc38a4* are imprinted in rats which, like humans, do not have this curious distribution pattern in oocytes [27, 28]. Therefore, the developmental relevance of “broad” peaks of histone modifications during the maternal-to-zygotic transition remains unclear. Furthermore, additional species-specific differences include the temporal regulation of PRC2-mediated H3K27me3. In mice, oocyte-derived PRC2-mediated deposition of H3K27me3 persists during pre-implantation development [25, 26], yet in humans, H3K27me3 is largely erased by the 2-cell stage, thus not providing the initial foundation epigenetic signal for non-canonical imprinting. Consistent with this, placenta cells preferentially inactivate the paternally inherited X chromosome in mice and rats, due to H3K27me3-associated non-canonical imprinting of *XIST* [22, 28], a signature we confirm is not observed in humans [48]. However, there are many, yet to be profiled, repressive heterochromatic marks in pre-implantation embryos that could facilitate non-canonical imprinting in human cleavage stage embryos. Involvement of an additional repressive histone modification, H3K9me2, has been suggested in mice since deletion G9a and/or GLP, the methyltransferases responsible for this mark, in growing oocytes results in upregulation of *Gab1* and *Sfmbt2* [13, 48–52]. Therefore, a comprehensive screening approach based on post-EGA monoallelic expression could reveal novel transiently imprinted genes that may not undergo the switch to maternal sDMR-mediated imprinting in the placenta. However, to categorically discount non-canonical imprinting in the human placenta would require an unbiased genome-wide screen for allelic expression across gestation in a cell-type specific manner. Whilst this is possible, challenges still remain in the fact that informative SNPs are often scarce and conclusion are based on a small number of informative loci/individuals. It is important to identify if these genes are present in the human genome at any developmental time point, as they may influence pre-implantation development and be subject to epigenetic instability if embryos are exposed to prolonged in vitro culture during assisted reproductive cycles.

## Material and methods

### Samples

A cohort of 32 control placenta samples with corresponding maternal blood/saliva samples were collected at the Hospital St. Joan De Déu (Barcelona, Spain) or Norfolk and Norwich University Hospital (Norwich, UK) to assess allelic expression and methylation. Both cohorts were collected using the same tissue preparation protocols in which multiple biopsies were taken from the fetal-side of the placenta, approximately 5 cm from the cord insertion site. All samples underwent microsatellite repeat analysis to confirm they were free of maternal DNA contamination.

All women had given written informed consent in accordance with the Declaration of Helsinki for themselves and their child prior to participating in the study. Ethical approval for collecting samples was granted by the Institutional Review Boards at Hospital St. Joan De Déu Ethics Committee (PI35/07) and the University of East Anglia Faculty of Medicine and Health Sciences Research Ethics Committee (ETH2122-0856).

The analysis of allelic expression in high quality human pre-implantation embryos (9 2–4 cells; 17 5–12 cells; 8 blastocysts) was performed by PCR on excess SMART-seq2 full-length cDNAs [53]. The use of materials from surplus embryos from assisted reproductive treatment cycles was approved by the scientific and ethic committee of the Instituto Valenciano de Infertilidad (IVI) for research protocols (1310-FIVI- 131-CS), University of East Anglia Faculty of Medicine and Health Sciences Research Ethics Subcommittee (ETH2223-1031), Bellvitge Institute of Biomedical Research, Barcelona (PR292/14), the Centro de Medicina Regenerativa de Barcelona (CMRB CEIC 10/2017), the National Committee for Human Reproduction (CNRHA) and the Regional Health Departments for Valencia and Catalyuna (4/2014 & 10/2017).

The human trophoblast cell lines CT^30^ and CT^mole#1^ were obtained from the Japanese Cell Repository. Cells were passaged using conditions described by Okae and colleagues [31, 32]. Wild type mouse embryos and placentas were produced by crossing C57BL/6 (B6) females with *Mus musculus molosinus* (JF1) male mice. RNA and DNA from cell lines and mouse tissues were isolated and extracted as previously described [54].

### Genotyping and imprinting analysis

We interrogated the hg19 genome build on the UCSC sequence browser to identify SNPs with MAF > 0.1. PCR primers were designed to flank the polymorphisms allowing genotype calling by direct sequencing. Sequence traces were assessed using Sequencher v4.6 or SnapGene v7.2 to distinguish heterozygous and homozygous samples. Heterozygous tissue samples were used for subsequent allelic RT-PCR, methylation-sensitive genotyping and bisulphite PCR (see Supplementary Material, Table S8 for primer sequence).

### Methylation-sensitive genotyping

Approximately 1 μg of heterozygous genomic DNA was digested with 10 units of HpaII (6 h at 37°C) or BstU1 (6 h at 60°C) restriction endonucleases (NEB). The digested DNA was subject to ethanol precipitation and resuspended in a final volume of 20 μl of water. Approximately 2.5 μl of digested DNA was used in each amplification reaction using Bioline Taq polymerase for 40 cycles. The resulting amplicons were sequenced and the sequence traces compared to those obtained for the corresponding undigested DNA and parental samples (see Supplementary Material, Table S8 for primer sequence).

### Bisulphite methylation analyses

Standard bisulphite conversion was performed using the EZ DNA Methylation-Gold kit (Zymo) following the manufacturer’s Alternative 2 instructions. Approximately 2.5 μl of bisulphite converted DNA was used in each amplification reaction using Immolase Taq polymerase (Bioline) for 45 cycles and the resulting PCR product sub-cloned into pGEM-T easy vector (Promega). Individual colony PCR was performed using primers flanking the multiple-cloning site and sequenced with T7 or M13F (see Supplementary Material, Table S8 for primer sequence).

### Analysis of expression

cDNA (for placenta) and full-length amplified cDNAs sequencing libraries (pre-implantation embryos) from heterozygous samples were subject to RT-PCR with direct sequencing of the resulting amplicons. Imprinting was suggested only if a single base peak was observed at the SNP site in the RT-PCR product of a heterozygous sample. The parental origin of expression was determined by phasing allelic expression with genotypes of blood or saliva-derived DNAs from biological parents. Whenever possible, RT-PCR primers were located in different exons, so that the PCR product crossed a splice site (see Supplementary Material, Table S8 for primer sequence). In addition, RT-PCR was performed on RT-positive and negative samples in order to rule out genomic contamination.

### Rapid amplification of cDNA ends (RACE) PCR

5’RACE-PCR was used to obtain full-length sequence for the *MBD3* mRNA transcript using the 5’/3’ RACE kit (Roche) as described previously [55].

### Bioinformatic analysis of Illumina EPIC array datasets

Probes mapping to regions of interest were extracted using in-house R scripts from published placenta cell-type specific methylation datasets published by Yuen *et al* (GEO159526) [56]. The EPIC array datasets for CT^30^ and CT^mole#1^ were generated in-house and data is available upon request.

## Supplementary Material

Supplemental_Data_Daskeviciute_HMG-2024-CE-00429_ddaf009

Supplementary_Table_S2_Daskeviciute_HMG-2024-CE-00429_ddaf009

Supplementary_Table_S5_Daskeviciute_HMG-2024-CE-00429_ddaf009

Supplementary_Table_S7_Daskeviciute_HMG-2024-CE-00429_ddaf009

## References

[1] McGrath J, Solter D. Completion of mouse embryogenesis requires both the maternal and paternal genomes. Cell 1984;37:179–183. 6722870 10.1016/0092-8674(84)90313-1

[2] Surani M, Barton S, Norris M. Development of reconstituted mouse eggs suggests imprinting of the genome during gametogenesis. Nature 1984;308:548–550. 6709062 10.1038/308548a0

[3] Ferguson-Smith A . Genomic imprinting: the emergence of an epigenetic paradigm. Nat Rev Genet 2011;12:565–575. 21765458 10.1038/nrg3032

[4] Moore T, Haig D. Genomic imprinting in mammalian development: a parental tug-of-war. Trends Genet 1991;7:45–49. 2035190 10.1016/0168-9525(91)90230-N

[5] Varmuza S, Mann M. Genomic imprinting--defusing the ovarian time bomb. Trends Genet 1994;10:118–123. 7848407 10.1016/0168-9525(94)90212-7

[6] Okae H, Chiba H, Hiura H. et al. Genome-wide analysis of DNA methylation dynamics during early human development. PLoS Genet 2014;10:e1004868. 25501653 10.1371/journal.pgen.1004868 PMC4263407

[7] Court F, Tayama C, Romanelli V. et al. Genome-wide parent-of-origin DNA methylation analysis reveals the intricacies of human imprinting and suggests a germline methylation-independent mechanism of establishment. Genome Res 2014;24:554–569. 24402520 10.1101/gr.164913.113 PMC3975056

[8] Bourc'his D, Xu G, Lin C. et al. Dnmt3L and the establishment of maternal genomic imprints. Science 2001;294:2536–2539. 11719692 10.1126/science.1065848

[9] Smallwood SA, Tomizawa S, Krueger F. et al. Dynamic CpG island methylation landscape in oocytes and preimplantation embryos. Nat Genet 2011;43:811–814. 21706000 10.1038/ng.864 PMC3146050

[10] Xu Q, Xiang Y, Wang Q. et al. SETD2 regulates the maternal epigenome, genomic imprinting and embryonic development. Nat Genet 2019;51:844–856. 31040401 10.1038/s41588-019-0398-7

[11] Dhayalan A, Rajavelu A, Rathert P. et al. The Dnmt3a PWWP domain reads histone 3 lysine 36 trimethylation and guides DNA methylation. J Biol Chem 2010;285:26114–26120. 20547484 10.1074/jbc.M109.089433 PMC2924014

[12] Ciccone D, Su H, Hevim S. et al. KDM1B is a histone H3K4 demethylase required to establish maternal genomic imprints. Nature 2009;461:415–418. 19727073 10.1038/nature08315

[13] Stewart K, Veselovska L, Kim J. et al. Dynamic changes in histone modifications precede de novo DNA methylation in oocytes. Genes Dev 2015;29:2449–2462. 26584620 10.1101/gad.271353.115 PMC4691949

[14] Demond H, Hanna C, Castillo-Fernandez J. et al. Multi-omics analyses demonstrate a critical role for EHMT1 methyltransferase in transcriptional repression during oogenesis. Genome Res 2023;33:18–31. 36690445 10.1101/gr.277046.122 PMC9977154

[15] Petrussa L, Van de Velde H, De Rycke M. Dynamic regulation of DNA methyltransferases in human oocytes and preimplantation embryos after assisted reproductive technologies. Mol Hum Reprod 2014;20:861–874. 24994815 10.1093/molehr/gau049

[16] Monk D, Morales J, den Dunnen J. et al. Recommendations for a nomenclature system for reporting methylation aberrations in imprinted domains. Epigenetics 2018;13:117–121. 27911167 10.1080/15592294.2016.1264561 PMC5873357

[17] Monk D . Genomic imprinting in the human placenta. Am J Obstet Gynecol 2015;213:S152–S162. 26428495 10.1016/j.ajog.2015.06.032

[18] Sanchez-Delgado M, Court F, Vidal E. et al. Human oocyte-derived methylation differences persist in the placenta7 revealing widespread transient imprinting. PLoS Genet 2016;12:e1006427. 27835649 10.1371/journal.pgen.1006427 PMC5106035

[19] Sanchez-Delgado M, Martin-Trujillo A, Tayama C. et al. Absence of maternal methylation in Biparental Hydatidiform moles from women with NLRP7 maternal-effect mutations reveals widespread placenta-specific imprinting. PLoS Genet 2015;11:e1005644. 26544189 10.1371/journal.pgen.1005644 PMC4636177

[20] Hanna C, Peñaherrera M, Saadeh H. et al. Pervasive polymorphic imprinted methylation in the human placenta. Genome Res 2016;26:756–767. 26769960 10.1101/gr.196139.115 PMC4889973

[21] Hamada H, Okae H, Toh H. et al. Allele-specific Methylome and transcriptome analysis reveals widespread imprinting in the human placenta. Am J Hum Genet 2016;99:1045–1058. 27843122 10.1016/j.ajhg.2016.08.021 PMC5097938

[22] Inoue A, Jiang L, Lu F. et al. Maternal H3K27me3 controls DNA methylation-independent imprinting. Nature 2017;547:419–424. 28723896 10.1038/nature23262 PMC9674007

[23] Okae H, Hiura H, Nishida Y. et al. Re-investigation and RNA sequencing-based identification of genes with placenta-specific imprinted expression. Hum Mol Genet 2012;21:548–558. 22025075 10.1093/hmg/ddr488

[24] Inoue A, Chen Z, Yin Q. et al. Maternal Eed knockout causes loss of H3K27me3 imprinting and random X inactivation in the extraembryonic cells. Genes Dev 2018;32:1525–1536. 30463900 10.1101/gad.318675.118 PMC6295166

[25] Mei H, Kozuka C, Hayashi R. et al. H2AK119ub1 guides maternal inheritance and zygotic deposition of H3K27me3 in mouse embryos. Nat Genet 2021;53:539–550. 33821003 10.1038/s41588-021-00820-3

[26] Chen Z, Djekidel M, Zhang Y. Distinct dynamics and functions of H2AK119ub1 and H3K27me3 in mouse preimplantation embryos. Nat Genet 2021;53:551–563. 33821005 10.1038/s41588-021-00821-2 PMC8092361

[27] Hanna C, Pérez-Palacios R, Gahurova L. et al. Endogenous retroviral insertions drive non-canonical imprinting in extra-embryonic tissues. Genome Biol 2019;20:225. 31665063 10.1186/s13059-019-1833-x PMC6819472

[28] Albert J, Kobayashi T, Inoue A. et al. Conservation and divergence of canonical and non-canonical imprinting in murids. Genome Biol 2023;24:48. 36918927 10.1186/s13059-023-02869-1 PMC10012579

[29] Wang Q, Chow J, Hong J. et al. Recent acquisition of imprinting at the rodent Sfmbt2 locus correlates with insertion of a large block of miRNAs. BMC Genomics 2011;12:204. 21510876 10.1186/1471-2164-12-204 PMC3110154

[30] Andergassen D, Smith Z, Kretzmer H. et al. Diverse epigenetic mechanisms maintain parental imprints within the embryonic and extraembryonic lineages. Dev Cell 2021;5:2995–3005. 10.1016/j.devcel.2021.10.010 PMC9463566 34752748

[31] Okae H, Toh H, Sato S. et al. Derivation of human trophoblast stem cells. Cell Stem Cell 2018;22:50–63.e6. 29249463 10.1016/j.stem.2017.11.004

[32] Takashasho S, Okae H, Kpobayashi N. et al. Loss of P57KIP2 expression confers resistance to contact inhibition in human androgenetic trophoblast stem cells. Proc Natl Acad Sci USA 2019;116:26606–26613. 31792181 10.1073/pnas.1916019116 PMC6936680

[33] Gendrel AV, Heard E. Noncoding RNAs and epigenetic mechanisms during X-chromosome inactivation. Annu Rev Cell Dev Biol 2014;30:561–580. 25000994 10.1146/annurev-cellbio-101512-122415

[34] Zhang W, Chen Z, Yin Q. et al. Maternal-biased H3K27me3 correlates with paternal-specific gene expression in the human morula. Genes Dev 2019;33:382–387. 30808660 10.1101/gad.323105.118 PMC6446541

[35] Brind'Amour J, Kobayashi H, Albert JR. et al. LTR retrotransposons transcribed in oocytes drive species-specific and heritable changes in DNA methylation. Nat Commun 2018;9:3331. 30127397 10.1038/s41467-018-05841-x PMC6102241

[36] Bogutz A, Brind'Amour J, Kobayashi H. et al. Evolution of imprinting via lineage-specific insertion of retroviral promoters. Nat Commun 2019;10:5674. 31831741 10.1038/s41467-019-13662-9 PMC6908575

[37] Conley A, Piriyapongsa J, Jordan I. Retroviral promoters in the human genome. Bioinformatics 2008;24:1563–1567. 18535086 10.1093/bioinformatics/btn243

[38] Monteagudo-Sánchez A, Sánchez-Delgado M, Guara Ciurana S. et al. Epigenetic symmetry of DLGAP2: pre-implantation maternal methylation switches to a random Monoallelic profile in somatic tissues. OBM Genetics 2018;2:1.

[39] Hon G, Rajagopal N, Shen Y. et al. Epigenetic memory at embryonic enhancers identified in DNA methylation maps from adult mouse tissue. Nat Genet 2013;45:1198–1206. 23995138 10.1038/ng.2746 PMC4095776

[40] Hanna C, Kelsey G. Features and mechanisms of canonical and noncanonical genomic imprinting. Genes Dev 2021;35:821–834. 34074696 10.1101/gad.348422.121 PMC8168557

[41] Noguer-Dance M, Abu-Amero S, Al-Khtib M. et al. The primate-specific microRNA gene cluster (C19MC) is imprinted in the placenta. Hum Mol Genet 2010;19:3566–3582. 20610438 10.1093/hmg/ddq272

[42] Barbaux S, Gascoin-Lachambre G, Buffat C. et al. A genome-wide approach reveals novel imprinted genes expressed in the human placenta. Epigenetics 2012;7:1079–1090. 22894909 10.4161/epi.21495 PMC3466192

[43] Kobayashi H, Yanagisawa E, Sakashita A. et al. Epigenetic and transcriptional features of the novel human imprinted lncRNA GPR1AS suggest it is a functional ortholog to mouse Zdbf2linc. Epigenetics 2013;8:635–645. 23764515 10.4161/epi.24887 PMC3857343

[44] Monk D, Arnaud P, Apostolidou S. et al. Limited evolutionary conservation of imprinting in the human placenta. Proc Natl Acad Sci USA 2006;103:6623–6628. 16614068 10.1073/pnas.0511031103 PMC1564202

[45] Soncin F, Natale D, Parast M. Signaling pathways in mouse and human trophoblast differentiation: a comparative review. Cell Mol Life Sci 2015;72:1291–1302. 25430479 10.1007/s00018-014-1794-x PMC4366325

[46] Xia W, Xu J, Yu G. et al. Resetting histone modifications during human parental-to-zygotic transition. Science 2019;365:353–360. 31273069 10.1126/science.aaw5118

[47] Liu X, Wang C, Liu W. et al. Distinct features of H3K4me3 and H3K27me3 chromatin domains in pre-implantation embryos. Nature 2016;537:558–562. 27626379 10.1038/nature19362

[48] Moreira de Mello J, de Araújo E, Stabellini R. et al. Random X inactivation and extensive mosaicism in human placenta revealed by analysis of allele-specific gene expression along the X chromosome. PLoS One 2010;5:e10947. 20532033 10.1371/journal.pone.0010947 PMC2881032

[49] Meng T, Lei W, Lu X. et al. Maternal EHMT2 is essential for homologous chromosome segregation by regulating cyclin B3 transcription in oocyte meiosis. Int J Biol Sci 2022;18:4513–4531. 35864958 10.7150/ijbs.75298 PMC9295060

[50] Albert J, Greenberg M. Non-canonical imprinting in the spotlight. Development 2023;150:dev201087. 37309910 10.1242/dev.201087

[51] Auclair G, Borgel J, Sanz L. et al. EHMT2 directs DNA methylation for efficient gene silencing in mouse embryos. Genome Res 2016;26:192–202. 26576615 10.1101/gr.198291.115 PMC4728372

[52] Yeung W, Brind’Amour J, Hatano Y. et al. Histone H3K9 methyltransferase G9a in oocytes is essential for preimplantation development but dispensable for CG methylation protection. Cell Rep 2019;27:282–293.e4. 30943408 10.1016/j.celrep.2019.03.002

[53] Hernandez Mora J, Buhigas C, Clark S. et al. Single-cell multi-omic analysis profiles defective genome activation and epigenetic reprogramming associated with human pre-implantation embryo arrest. Cell Rep 2023;42:112100. 36763500 10.1016/j.celrep.2023.112100

[54] Maupetit-Méhouas S, Montibus B, Nury D. et al. Imprinting control regions (ICRs) are marked by mono-allelic bivalent chromatin when transcriptionally inactive. Nucleic Acids Res 2016;44:621–635. 26400168 10.1093/nar/gkv960 PMC4737186

[55] Monteagudo-Sánchez A, Sánchez-Delgado M, Mora J. et al. Differences in expression rather than methylation at placenta-specific imprinted loci is associated with intrauterine growth restriction. Clin Epigenetics 2019;11:35. 30808399 10.1186/s13148-019-0630-4 PMC6390544

[56] Yuan V, Hui D, Yin Y. et al. Cell-specific characterisation of the placenta methylome. *B.M.C*. Genomics 2021;22:6. 33407091 10.1186/s12864-020-07186-6 PMC7788826

