# Supplementary material for: Non-canonical imprinting, manifesting as post-fertilization placenta-specific parent-of-origin dependent methylation, is not conserved in humans
Source: Hum Mol Genet. 2025 Jan 17;34(7):626–38. doi: 10.1093/hmg/ddaf009 (PMC11924184; doi:10.1093/hmg/ddaf009)
Supplement: Supplemental_Data_Daskeviciute_HMG-2024-CE-00429_ddaf009 [file supplemental_data_daskeviciute_hmg-2024-ce-00429_ddaf009.pdf]

Supplementary Material

Supplementary Figure 1. Quantitative RT-PCR comparing the expression of candidate non-canonical imprinted genes in human trophoblast stem cells of biparental (CT^{30}) **and androgenetic (**$CT^{mole\#1}$**) origin.** All expression is relative to CT^{30} values and normalised to the average of *ACTB* and *RPL19* housekeeping gene.

Supplementary Figure 2. Overview of the methylation-sensitive genotyping assay. (A) Schematic overview of the methylation-sensitive HpaII genotyping assay. (B) The resulting PCR profiles for loci that are unmethylated (*KFL10* promoter), an imprinted gDMR (*SNURF*), fully methylated on both parental alleles (CpG83 of *RASSF1*) and random monoallelically methylated (*DLGAP2*). (C) The resulting Sanger sequencing of heterozygous DNA samples. (D) Confirmation bisulphite PCR and sub-cloning of the across the same SNPs. Each circle represents a single CpG on the strand. (•) methylated cytosines, (o) unmethylated cytosines. Each row corresponds to an individual clones sequence. The parental inheritance pattern for each genotype is incorporated into the amplicon.

Supplementary Figure S3. Analysis of allelic methylation, using methylation-sensitive genotyping for candidate sDMRs in placenta. Representative sequence traces of PCR products generated using *Hpa*II digested DNA in heterozygous placenta samples are shown for *LTK, C5ORF38, FFAR1, PLXDC1, ECRG4, SULF1* and *KRT86.*

Supplementary Figure S4. Characterization of eight novel maternally methylated placenta- specific gDMRs. (A) Representative sequence traces of PCR products generated using *Hpa*II digested DNA in heterozygous placenta samples are shown for *DYRK1B*,

LRRC8D, *WNT7B*, *CLDN23*, *WNT7B*, *PRKAG2* and *STARD13* for which methylation is restricted to maternal alleles.

Supplementary Figure S1

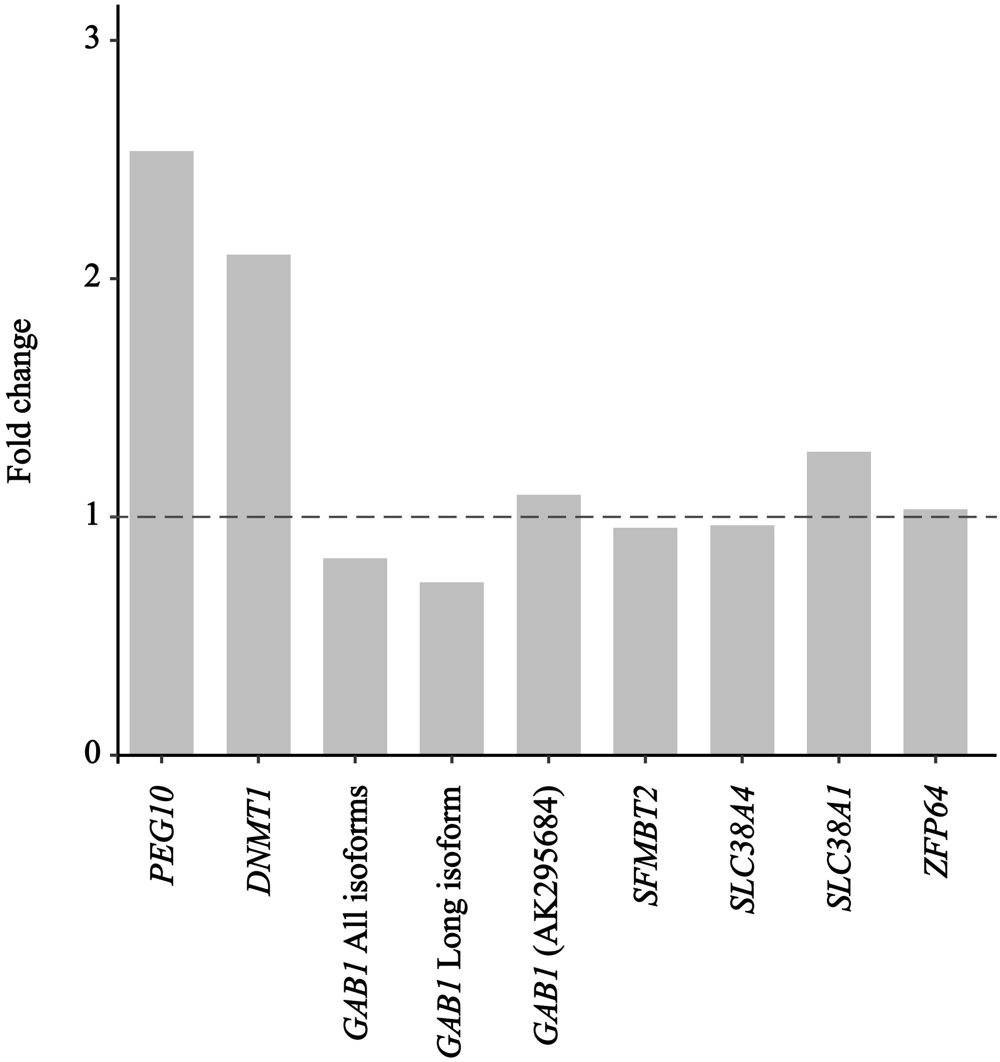

Supplementary Figure S2

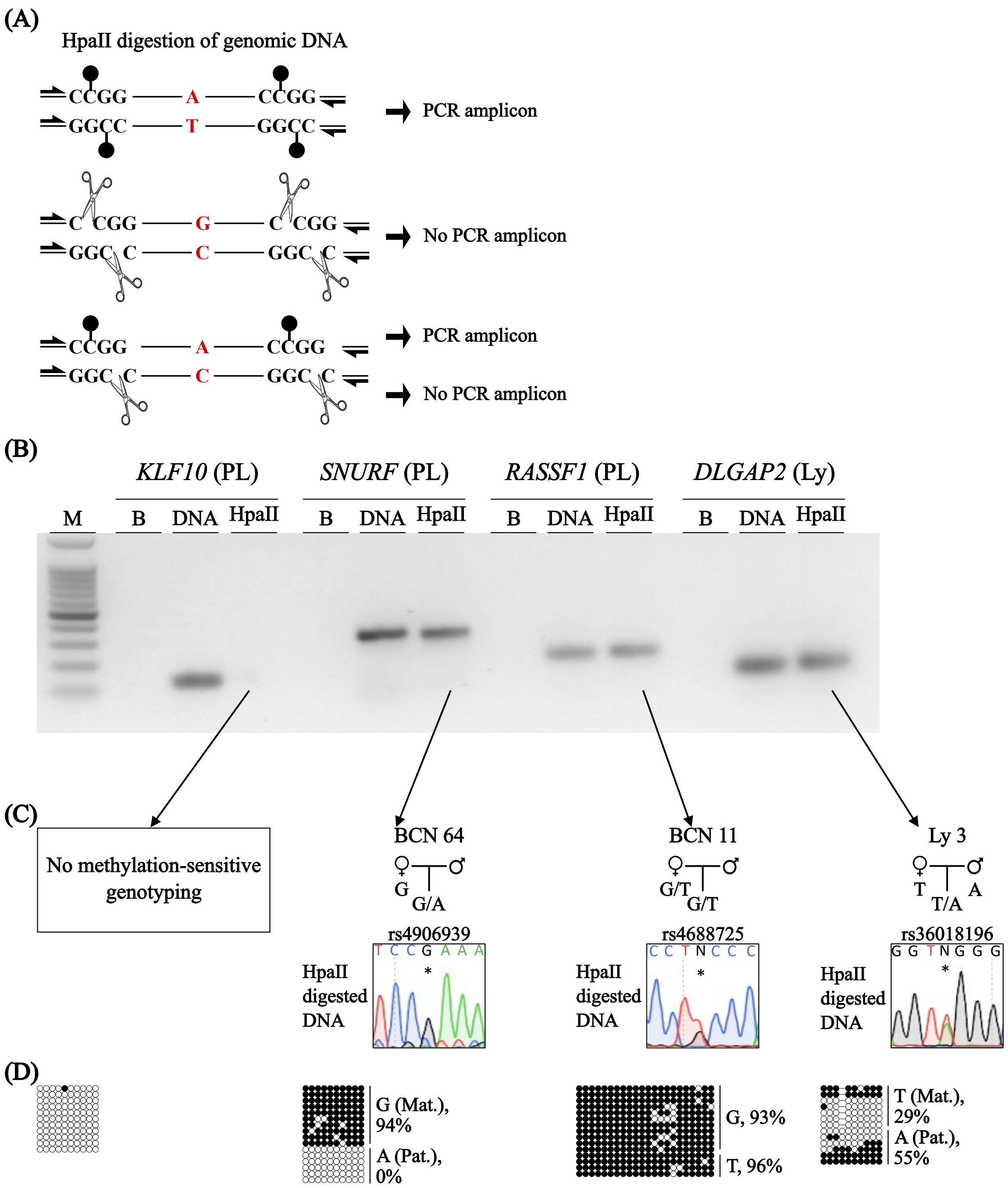

Supplementary Figure S3

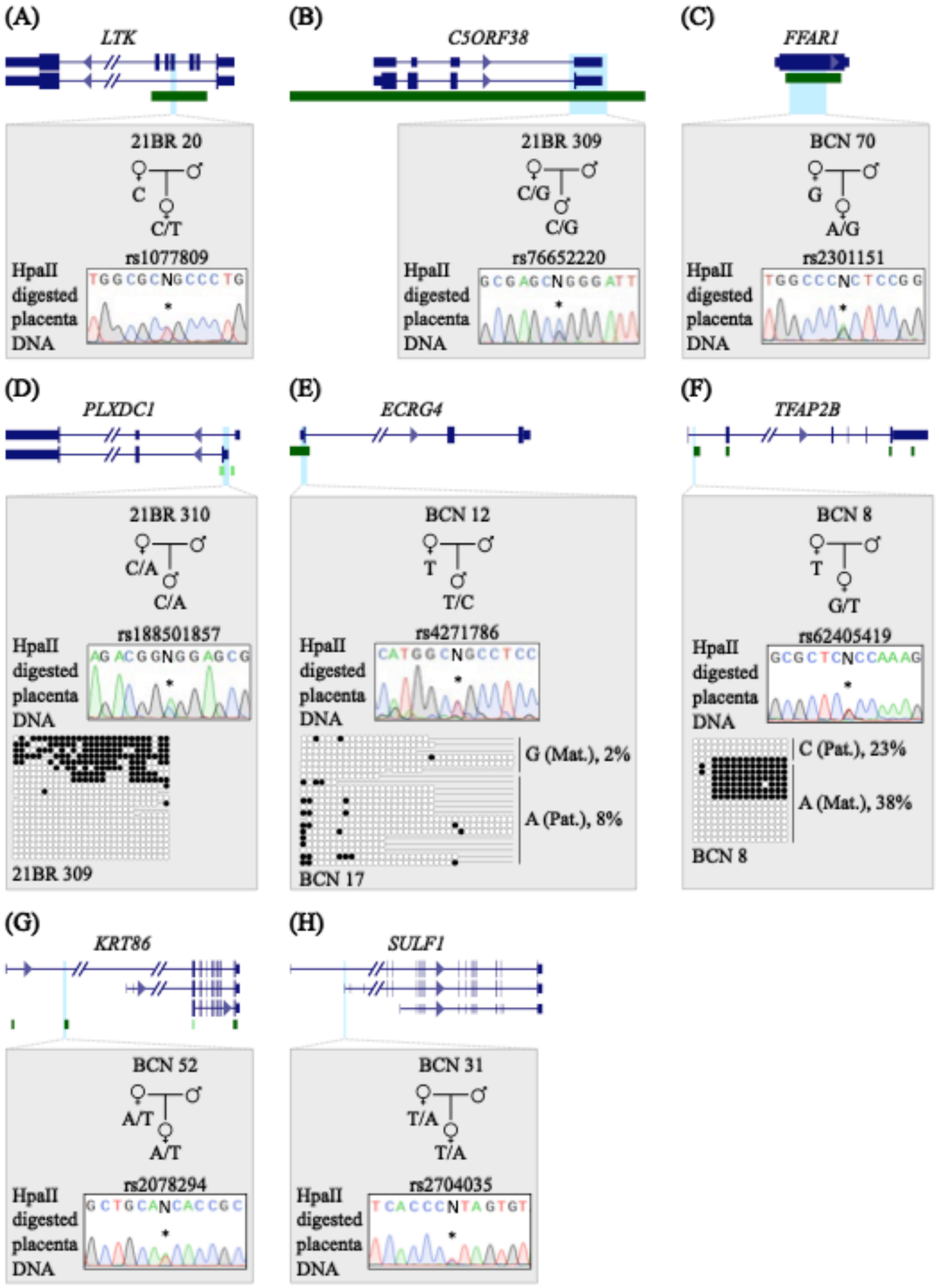

Supplementary Figure S4

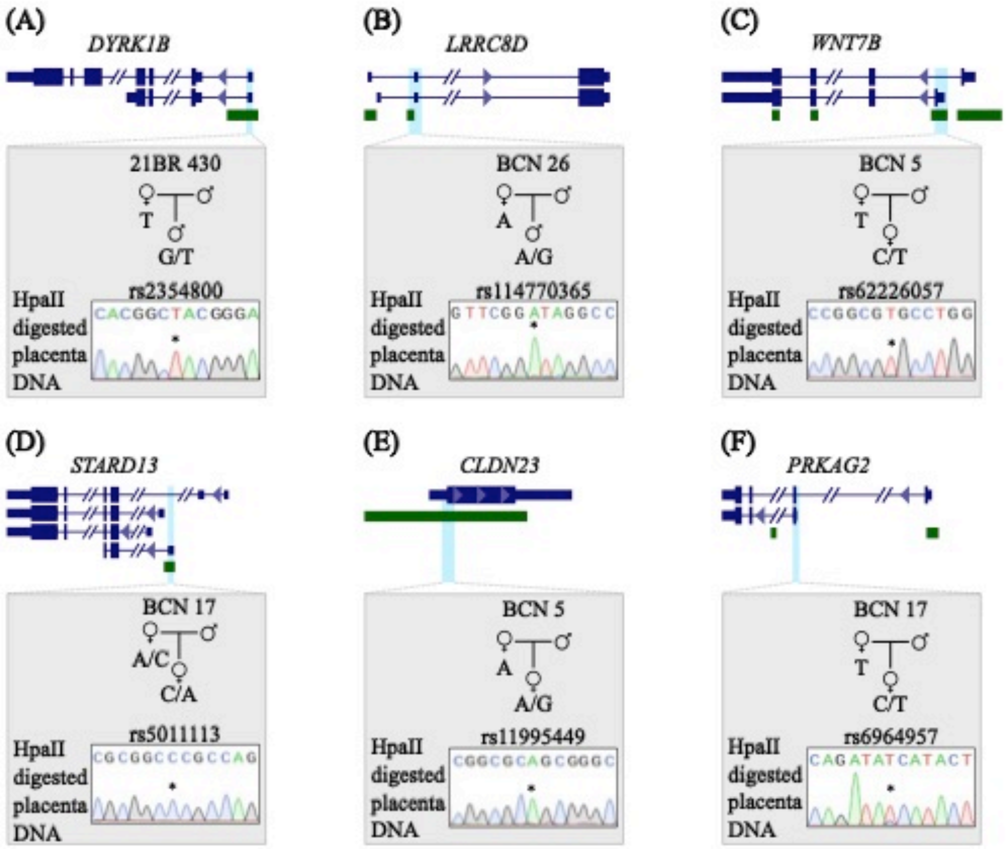

Supplementary Table S1. The number of heterozygous placenta samples used to determine allelic methylation and expression for non-canonical imprinted genes in mouse and rat.

**Chr.**	**Gene**	**SNP**	**Number of heterozygous samples**	**Methylation-sensitive genotyping (HpaII & BstUI)**	**Allelic RT-PCR**	**Methylation - cloning**
3	*RPL39L*	-	-	-	-	BCN 8 - not informative, low, mosaic methylation
3	*RPL39L*	rs141173382	1	-	-	BCN 8 - informative, both alleles mostly methylated
4	*JADE1/ PHF17*	rs62317870	-	-	-	BCN 26 & BCN 17 - not informative, low methylation
4	*JADE1/ PHF17* (NM_001287441; NM_024900)	rs13114904	4	-	4 - biallelic	-
4	*JADE1/ PHF17* (NM_001287437)	rs11933240	4	-	4 - biallelic	-
4	*GAB1*	-	-	-	-	BCN 6 (NM_002039) - not informative, unmethylated
4	*GAB1*	rs1397529	4	-	4 - biallelic	-

4	*GAB1* (AK295684)	rs62337524	1	-	1 - biallelic	21BR 309 - informative, mosaic methylation
10	*SFMBT2*	-	-	-	-	BCN 8 - not informative, unmethylated
		rs10795530	4	-	1 - pref. monoallelic, 3 - biallelic	-
12	*SLC38A1*	-	-	-	-	BCN 8 - not informative, mosaic methylation
		rs1045278	6	-	6 - biallelic	-
12	*SLC38A4*	-	-	-	-	BCN8 - not informative, mostly unmethylated
		rs4994910	10	6 - pref. monoallelic, 4 - biallelic	-	-
		rs74851348	3	1 - monoallelic, 1 - pref. monoallelic, 1 - biallelic	-	BCN 8 - not informative, mostly methylated; 21BR 309 - not informative mosaic methylation; 21BR 19 - informative, mosaic methylation,

						mostly methylated
		rs2429467	3	-	3 - biallelic	-
14	*SMOC1*	rs146095118	-	-	-	BCN 26 - not informative, low methylation
		rs3742909	4	-	4 - biallelic	-
16	*SALL1*	rs11643654	1	-	-	21BR 307 - not informative, mosaic methylation; BCN 5 - informative, mosaic methylation
		rs11645288	4	-	4 - biallelic	-
18	*ZNF516*	-	-	-	-	BCN 8 - not informative, mosaic methylation
		rs690353	1	-	1 - biallelic	-
20	*ZFP64*	-	-	-	-	BCN 8 - not informative, mosaic methylation
		rs3746413	2	-	2 - biallelic	-
X	XIST	-	-	-	-	Male sample fully methylated, female sample

					showed 50% methylation
	rs1894271	2	-	2 - biallelic	-

Supplementary Table S2 – see excel tables. Illumina EPIC array methylation profiles for candidate intervals for placenta villi, immune-selected trophoblasts, endothelial, stromal and Hofbauer cells. The methylation is presented as the average Beta-value for 19 term samples in immune-selected cells types. Data taken from GEO159526. Each table also include Beta-value for CT^{30} and $CT^{mole\#1}$ cell lines.

Supplementary Table S3. The number of heterozygous preimplantation embryos used to determine allelic expression.

Chr.	Gene	SNP	Embryo stage	Allelic expression
4	*JADE1*	rs11933240	Blastocyst	1 (TE) - biallelic, 1 (ICM) - biallelic, 1 (whole embryo) - biallelic
4	*GAB1*	rs1397529	Cleavage stage 2	1 - biallelic
			Cleavage stage 3	2 - biallelic
			Blastocyst	1 (TE) - biallelic, 2 (ICM) - biallelic
	GAB1	rs1360288278	Cleavage stage 2	1 - biallelic
	GAB1	rs28924077	Morula	1 - biallelic
12	*SLC38A1*	rs1045278	Blastocyst	1 (TE) - biallelic, 2 (whole blastocysts) - biallelic, 1 (whole blastocyst) - pref. monoallelic
		rs3498	Blastocyst	1 (whole blastocyst) - biallelic
		rs61923106	Blastocyst	1 (whole blastocyst) - biallelic
		rs1938843414	Blastocyst	1 (whole blastocyst) - pref. monoallelic
18	*ZNF516*	rs72973711	Blastocyst	1 (TE) - biallelic, 1 (whole embryo) - biallelic
		rs2074488845	Blastocyst	1 (TE) - pref. monoallelic, 1 (whole embryo) - pref. monoallelic

Supplementary Table S4. The number of heterozygous placenta samples used to determine allelic methylation for LTR-derived transcripts.

Chr.	Gene	SNP	Number of heterozygous samples	Methylation-sensitive genotyping (HpaII & BstUI)	Allelic RT-PCR	Methylation - cloning
1	*ERO1B*	rs557205	3	3 - fully digested	-	-
		rs73117239	3	3 - fully digested	-	-
2	*GALNT13*	rs62174125	6	2 - pref. monoallelic, 3 - biallelic, 1 - fully digested	-	BCN 8 & BCN 5 - not informative, low, mosaic methylation
		rs12999856	6	1 - monoallelic, 3 - biallelic, 2 - fully digested	-	BCN 8 - not informative, mosaic methylation; BCN 5 - informative, low, mosaic methylation
		rs10194599	5	2 - monoallelic, 2 - biallelic, 1 - fully digested	-	-
2	*SCHLAP1*	rs144415983	2	1 - pref. monoallelic, 1 - biallelic	-	21BR 21 - informative, mosaic methylation
		rs148398319	2	2 - monoallelic	-	21BR 21 - not informative, mosaic methylation
		rs7560378	3	1 - pref. monoallelic, 2 - biallelic	-	21BR 21 - not informative, mosaic methylation
4	*SLC7A11 - AS*	rs7693285	4	1 - pref. maternal (HpaII), 3 - biallelic (HpaII);<br>2 - pref. maternal (BstUI), 1 - pref.	-	BCN 8 - informative, mosaic methylation

				monoallelic (BstUI), 1 - biallelic (BstUI)		
		rs7699108	4	1 - pref. maternal (HpaII), 3 - biallelic (HpaII); 2 - pref. maternal (BstUI), 1 - pref. monoallelic (BstUI), 1 - biallelic (BstUI)	-	BCN 8 - not informative, mosaic methylation
17	*LOC339166*	rs12450161	9	2 - maternal, 2 - paternal, 4 - monoallelic, 1 - pref. monoallelic	-	-
		rs12450165	9	3 - maternal, 2 - paternal, 4 - monoallelic	-	-
		rs12453225	9	2 - pref. maternal, 1 - pref. paternal, 4 - pref. monoallelic methylation, 2 - biallelic	1 - maternal, 3 - monoallelic, 1 - pref. maternal, 1 - pref. paternal, 1 - pref. monoallelic, 2 - biallelic	21BR 430 - informative, low, mosaic methylation

Supplementary Table S5– see excel tables. The approximately size and CpG density of human ubiquitous and placenta-specific gDMRs, as well as mouse and rat non-canonical sDMRs as determined by placenta methyl-seq.

Supplementary Table S6. The number of heterozygous placenta samples used to determine allelic methylation and expression at candidate non-canonical imprints.

Chr.	Gene	SNP	Number of heterozygous samples	Methylation-sensitive genotyping (HpaII & BstUI)	Allelic RT-PCR	Methylation - cloning
1	*DNAJC6*	rs577841	6	1 - monoallelic, 1 - pref. maternal, 2 - pref. monoallelic, 2 - biallelic methylation	-	-
2	*C2ORF40 & ECRG4*	rs4271786	13	1 - maternal, 1 - paternal, 1 - pref. paternal, 4 - pref. monoallelic, 5 - biallelic	-	BCN 17 - informative, both alleles mostly unmethylated
		rs4266035	13	1 - maternal methylation, 1 - paternal, 3 - pref. monoallelic, 7 - biallelic	-	BCN 17 - informative, both alleles mostly unmethylated
		rs73949223	1	1 - pref. monoallelic	-	-
		rs4477942	9	1 - maternal, 1 - pref. monoallelic, 7 - biallelic	-	-
4	*CRMP1*	rs139357095	2	2 - biallelic	-	-
4	*CWH43*	rs3747690	14	1 - monoallelic,	-	-

				1 - pref. maternal, 1 - pref. paternal, 1 - pref. monoallelic, 8 - biallelic, 2 - fully digested		
5	*C5ORF38*	rs62333235	2	2 – biallelic	-	-
		rs76652220	3	3 - biallelic	-	-
5	*ANKDD1B*	rs72633976	6	1 - paternal, 4 - monoallelic, 1 - pref. monoallelic	-	-
		rs1489	2	1 - monoallelic, 1 - pref. monoallelic	-	-
		rs61516153	2	2 - monoallelic	-	-
6	*TFAP2B*	rs4628086	1	1 - biallelic	-	BCN 8 - not informative, low methylation
		rs62405419	3	3 - biallelic	-	BCN 8 - informative, both alleles methylated, low methylation, maternal allele more methylated
8	*SULF1*	rs2704035	4	4 - biallelic	-	-

8	*RGS22*	rs2453627	12	1 - pref. monoallelic, 11 - biallelic	-	-
12	*KRT86*	rs2078294	2	2 - biallelic	-	-
12	*FAM101A* (all isoforms)	rs12318072	7	-	2 - pref. monoallelic, 5 - biallelic	-
12	*FAM101A* (NM_001204299)	rs12823740	1	-	-	21BR 308 - informative, unmethylated
12	*FAM101A* (NM_001365156)	-	-	-	-	BCN 5 - not informative, low, mosaic methylation
		rs12318072	5	-	3 - maternal, 2 - biallelic	-
12	*FAM101A* (NM_181709)	rs12318072	6	-	1 - pref. maternal, 1 - pref. monoallelic, 4 - biallelic	-
15	*LTK*	rs1077809	4	4 - biallelic	-	-
17	*PLXDC1*	rs188501857	4	1 - monoallelic, 1 - pref. monoallelic, 2 - biallelic	-	21BR 309 - not informative mosaic methylation, lower

						methylation; BCN 50 - not informative mosaic methylation.
19	*NUDT19*	rs8108621	6	1 - maternal, 1- paternal, 1 - monoallelic, 3 - biallelic	1 - pref. maternal, 1 - pref. monoalleli c, 1 - biallelic	BCN 17 - not informative, mosaic methylation
		rs8109823	6	1 - maternal, 1 - paternal, 1 - pref. maternal, 1 - monoallelic, 2 - biallelic	1 - pref. maternal, 2 - biallelic	BCN 17 - not informative, mosaic methylation
		rs61732600	11	3 - monoallelic, 3 - pref. monoallelic, 5 - biallelic	2 - biallelic	BCN 17 - informative, mosaic methylation, paternal allele more methylated
19	*FFAR1*	rs2301151	8	1 - pref. maternal, 1 - pref. monoallelic, 6 - biallelic	-	-
20	*TSPY26P & PLAGL2*	rs11907716	9	1 - maternal, 7 - monoallelic, 1 - biallelic	-	-
		rs11907235	12	2 - maternal, 2 - paternal,	-	-

			8 -		
			monoallelic		

Supplementary Table S7– see excel tables. List of 94 candidate genes with germline, embryo and placenta methylation profiles consistent with non-canonical sDMRs.

Supplementary Table S8. The number of heterozygous placenta samples used to determine allelic methylation and expression at novel gDMRs.

Chr.	Gene	Comments	Variants	Oligo name	Sequence (5’ -> 3’)
**Genotyping (DNA)**					
1	*LRRC8D*	-	rs114770365, rs115363384, rs114208181, rs113834473	Forward	TCTATAACGTGCTGCCGGGTCT
				Reverse	CAGCTCCAGCGCAGCCCGGGGC
1	*ERO1B*	-	rs557205, rs73117239	Forward	AAACGAAACGAAGCCAAACAGA
				Reverse	CGGTGTCAGTGTGACTACATTTCC
				Sequencing Reverse	GTGTTACATTTACATAGTGG
2	*ECRG4/ C2ORF40*	-	rs4271786, rs4266035, rs73949223, rs4477942	Forward	GAGAGAGGACCTCGGTGGTACT
				Reverse	CACCCCATCACCGATCGCTCT
				Sequencing Forward	GGCAGCGACGCAGGGATAAC
2	*GALNT13*	-	rs62174125, rs12999856, rs10194599	Forward	TTGATCTGAGGCTGAATCCCGT
				Reverse	CAGAAAGTTCCGCGCCACGCGGTC
2	*SCHLAP1*	-	rs144415983, rs148398319, rs7560378	Outer Forward	CACTCACCGCGAAGGTCCGCAGC
				Outer Reverse	TTTCAGTCTGACCAATCAGGAGT
				Inner Forward	GAGGAACGAACAACTCCCGAC
				Inner Reverse	CAGCCAGCACAGTGTTCACCTAGA
3	*RASSF1*	-	rs4688725	Forward	ATGCGCAGCGCGTTGGCACGCTCCA

				Reverse	GATCCTGGGGGAGGCGCTGAAG
4	*CRMP1*	-	rs139357095	Forward	GTACCTGGCCATTGTCCCGGCCGAG
				Reverse	AGGGCGCCTACGAGAACAAGACCA
4	*CWH43*	-	rs3747690	Forward	AGGAGGCAAAGGCGGGACCAGA
				Reverse	CAAGAGGATTTCTCTCCACAGC
4	*JADE1*	NM_001287437	rs11933240	Forward	AGGAGAAGCATCTTGGCTTCTTGA
				Reverse	CAAATAACACAAACTTCTCAC
		NM_001287441, NM_024900	rs13114904	Forward	AGGCTGGAGTGCAGTGGCGTGA
				Reverse	CAAATAACTGCAACTCTCTGGGC
				Sequencing Forward	TGAGTAGCTGGGATTACAGGCGT
4	*SLC7A11-AS1*	-	rs7693285, rs7699108	Forward	TCACTGCCCGGTGCTTGCGGGCT
				Reverse	GTTAAAACAAATACTTCTTCG
4	*GAB1*	-	rs1397529	Forward	AGATGAATTGTAGACTAGTAACA
				Reverse	GATAGTTTAGGCACATTTCAGG
		AK295684	rs62337524	Forward	TTAGAAGCCTGCCCCAGAGTCT
				Reverse	CTTCTCTGTACCTCTGACTTC
5	*C5ORF38*	-	rs62333235, rs76652220	Forward	TCAGGAGTCGCTTAGGTTTT
				Reverse	GGCAGTTTCAGGTTCCTGGTG
5	*ANKDD1B*	-	rs72633976, rs1489, rs61516153	Forward	CAGGTCTTCCCTGAGACCCTT
				Reverse	GATTATCCCAGGCCAGCCCAAGTC
6	*TFAP2B*	-	rs4628086, rs62405419	Forward	TAGCAGTTTATTAGTTTCTGTTTTCT
				Reverse	GGAGCCGTCTGGCCGCGTCAG
7	*PRKAG2*	5'UTR/ DMR	rs6964957	Forward	CCCATCCCTGCAGAGTGCAC
				Reverse	GCCTGGTTTCTGAACTTCATAG
		Exonic SNP	rs8961	Forward	ACAAAAGGAGACAGAAACGGA
				Reverse	CAACATCACTGGAAGAAATAC
8	*CLDN23*	-	rs9644774, rs11995449	Forward	TGACTTCGGGTCCCCGGAGCCT
				Reverse	GTCCACTGGCTGGTTCAGGAAG
8	*DLGAP2*	-	rs36018196	Forward	CCTCGGACCACCTCCAAGCC
				Reverse	TGGTCCGCACAGAGCTGCTCT
8	*KLF10*	-	-	Forward	GACAAGACCAGGCGAGGAAG
				Reverse	GTGGCCCCGCGAGCCCATTGG

8	*SULF1*	-	rs2704035, rs2725092	Forward	AGTTTGTTTGCCGAGGTTTGCA
				Reverse	CTCTGATCCTCGCTGCCCTCGC
8	*RGS22*	-	rs2453627	Forward	ACCCCCAGCGCGGTCACCCGGAA
				Reverse	GCATTTCATACAACTGTGATG
10	*SFMBT2*	-	rs10795530	Forward	AGGTTGTAATGCAGTGGCGCA
				Reverse	CAGATGTTCTAGGCTTCAATC
				Sequencing Forward	TCTCAGCTCACTGCAACCTCT
		LTR	rs719809	Forward	CACAGGCATAAGCTATTAAC
				Reverse	AGACTAATATAGATAATGTAGA
10	*GSTO1*	-	rs4925	Forward	TCCTTGGTAGGAAGCTTTAT
				Reverse	GAATTTTCCTAATTACCTTAAAG
12	*SLC38A1*	-	rs1045278	Forward	CATCCCTGTTGTCTGCTCAGTC
				Reverse	ATATGATTGTATGAAATTTGAAAAA
12	*SLC38A4*	Exonic SNP	rs2429467	Forward	GTTCTGAACATCAACACAAAG
				Reverse	TGCTCATTGCTGCCTTTTCT
				Sequencing Forward	GGAAGAACCTTAAGCTGAAGG
		Upstream 5' UTR/ CpG island	rs4994910, rs74851348	Forward	GCCACCTCTCCTGGACTCAAGGGTG
				Reverse	AAGGGAGAAGGCGAGAGCAGA
				Sequencing Reverse	CGGTTCCGAGGGCGGCTTAC
12	*KRT86*	-	rs2078294	Forward	GTGAGGCCGCGGTAGCAGGAG
				Reverse	TGGCTCGCTTTCATTCCCGGCT
12	*EID3*	-	rs7488680, rs58078551	Forward	CCAAACACCACCTTGCAAAAGAAC
				Reverse	AAGTGGCGGCAGTTAGAGCCGA
12	*FAM101A*	-	rs12318072	Forward	CAACTCTGAGGTCAAGTACGCC
				Reverse	TCCGGAAAGTGCTCCTGGCAT
				Sequencing Reverse	GGCATGCTTGGGGAAGATG
13	*STARD13*	DMR / 5' UTR/ CpG island	rs5011113	Outer Forward	CAGCGGAGCAGGGGACAGCC
				Outer Reverse	CAGGTCAGTGCCCCGGAGAC

				Inner Forward	CAGCCCCTCCAGGTAACCCGTC
				Sequencing Inner Reverse	AGGGCATGAGTTTCAGAGCCCA
		Exonic SNP	rs495680	Forward	CTCCAGAATTCGCCGCCACC
				Reverse	ATCCTTGGGAATATGCACCA
14	*SMOC1*	-	rs3742909, rs146095118	Forward	TTCTAATAAGTGACCGTGAC
				Reverse	CATCTACCTCGATGCACCACGC
		Line	rs72725801	Forward	GTTTAGAAGTTATGTCCCACTG
				Reverse	TTTCAGCTGTCAGCTGGGGCT
15	*LTK*	-	rs1077809	Forward	CCCACTGGCTGCGCTCACTCC
				Reverse	TAGCCCTTACCCGGAACCTCTT
15	*SNURF*	-	rs4906939	Forward	ACTGCGCCACAACCGGAAAGGA
				Reverse	GTAGAGCCGCCAGTGGGGAGG
16	*SALL1*	-	rs11645288	Forward	GCTGATGACTCTGGGGGCATG
				Reverse	TGTGGCAAAACCTTCTCCTCAT
17	*LOC339166*	-	rs12450161, rs12450165, rs12453225	Forward	CTCCAGACGCGCCGCCTTAAG
				Reverse	ATATGGAGGGACTGCCCTGTAGA
17	*PLXDC1*	-	rs188501857	Forward	CCCGCCAGTCCTACCTGCTCC
				Reverse	TCGCGCTCTCGCCGCTCCT
18	*ZNF516*	-	rs690353	Forward	ACATCTTACCTCTGTGCTCCA
				Reverse	CGTCCTACACTCCATCAAAC
19	*MDB3*	DMR/ close to CpG island/ downstream 5' UTR	rs8104174	Forward	TGGCACCAATACCCTGCACATT
				Reverse	CCAGGCCGGACTGCATATCC
		Exon SNP	rs190802753	Forward	AGGAGGAGGAGCCCGACCCGGA
				Reverse	GCCAGGAGCACGGCCTTCCTCCTG
19	*NUDT19*	-	rs8108621, rs8109823, rs61732600	Forward	AAGGCTTCATGCCGGGCGCGCA
				Reverse	CGCAGGAAGTGGCGCGGGTCC
19	*DYRK1B*	-	rs2354800	Forward	TCCCTTGGCCTCGTGCTAAGTCT
				Reverse	GGGGCGGAGTCCAGGGCGTGG

20	*TSPY26P*	-	rs11907716, rs11907235	Forward	TCTTGAAGATGGCGCCCTCCTCCT
				Reverse	CAGAGGCTCCCGCAGGCGATGGC
				Sequencing Reverse	GAGGGCGGGGACCCCAGAAG
20	*ZFP64*	-	rs3746413	Forward	GTCGGAGCATCCTGAGAAGTG
				Reverse	TCTAGAGCCTCAGTCTTAACCAT
22	*WNT7B*	-	rs62226057	Forward	GAGCCTGTTCAGCCCCGCCAG
				Reverse	GTGCTCCACCTCGGCAGCTTAG
X	*XIST*	-	rs1894271	Forward	TGAAGGACAGCATGGTTGGT
				Reverse	CACATGGAATGAGCAGTGTGC
**Bisulphite PCR**					
2	*ECRG4/ C2ORF40*	-	rs4271786, rs4266035	Outer Forward	GGTTTTAGTATAGGAGTAGGAGTAG
				Outer Reverse	TTAACCTTAAAACCCAAAAACT
				Inner Forward	GGTTAGGGTTAGGATAGTAGG
				Inner Reverse	CTTAACCCTCAACCCTCTAA
2	*GALNT13*	-	rs62174125, rs12999856	Outer Forward	GGGGTTGGTYGAGGTTGGA
				Inner Forward	GGGTAAGTGTGAAGAGAGAGG
				Reverse	AAAACCTACTATCCTAACCA
2	*SCHLAP1*	-	rs144415983, rs148398319, rs7560378	Outer Methylated Forward	GAGTTGTAATATTTATCGCGAAGG
				Outer Unmethylated Forward	GAGTTGTAATATTTATTGTGAAGG
				Outer Reverse	ACTCCTATAACTAATTTATATTCTCA
				Inner Forward	GAGTTTATTGGGAGGAA

				Inner Reverse	AAAAAACTCACCCTAAAAACTTA
3	*RASSF1*	-	rs4688725	Forward	GTTTTAGATGAAGTCGTTATAGAG
				Reverse	TAAACTACGAAAACTAACACCC
3	*RPL39L*	LTR promoter (MER4E1, MER61C)	rs141173382	Outer Forward	GTGTAAGTTATAGGGGATGTGATG
				Outer Reverse	ACATATTCAATATAAACCAACCA
				Inner Forward	GTTTGGTTTGGGTTTAGAGGTTTG
				Inner Reverse	AACCCTAACTACATTATCTACA
		Promoter/ 5' UTR	-	Outer Forward	GTTGTTTAGTTGTTGTTTGG
				Inner Forward	GTTGTTTGGTTAYGGTATTTAG
				Reverse	AACCCAAACTATAAACCTCTAA
4	*JADE1*	-	rs62317870	Outer Forward	ATTAGTTTYGGTGTAGTGA
				Outer Reverse	CTACCRCTCCCATCTTAAAAC
				Inner Forward	TTTTATTTGAAAGTGGTTATTT
				Inner Reverse	TTTAATTTACAACTAAAACC
				Outer Forward 2	TTTTATTTGAAAGTGGTTATTT
				Outer Reverse 2	CTTCCTATAACAAAAATAATAC
				Inner Forward 2	GGTTTTAGTTGTAAATTAAA
				Inner Reverse 2	ACAATTTCCAAAACTATC
4		-		Forward	AGTTTTTTATTGTTYGGTGTTTG

	SLC7A11-AS1		rs7693285, rs7699108	Inner Reverse	CTTACCAAACAAAATAAATTCC
				Outer Reverse	ATAAATTAATACCAATTCCTATTAAA
4	*GAB1*	AK295684 promoter	rs62337524	Outer Forward	GAATAGTTTTGGGAGGTGG
				Outer Reverse	TAACCTAACCTACACCCAAAT
				Inner Forward	GTTATAGGGAGGATTATTTG
				Inner Reverse	ATAACTTCAACTACTCCACATTA
		Major promoter	-	Outer Forward	TGGAGTTTGTTYGTTTAGTT
				Outer Reverse	CAACTCTACTTACATAAC
				Inner Forward	ATTAGGAGAGTTAGGTTTT
				Inner Reverse	AAACAAACCACTTCACCACC
6	*TFAP2B*	-	rs4628086, rs62405419	Outer Methylated Forward	GTTCGAGTCGGAAAAGGGTTTTG
				Outer Unmethylated Forward	GTTTGAGTTGGAAAAGGGTTTTG
				Outer Reverse	ACTTCCTTAAAAATCACTA
				Inner Reverse	CCTCCTATATAAACATCTTTCA
8	*DLGAP2*	-	rs36018196	Forward	AGTAAGATTTTGTGTTGGAGAAAGTTAYG
				Reverse	CRTCCTTATCRAACAAAAACCRG
8	*KLF10*	-	-	Forward	AGGAAGTATAGGGGTATTTTTAAATGA
				Reverse	CTCACACACCTTTACCGTTAATTAAC

10	*GSTO1*	-	-	Outer Forward	TGTAAATTTTAGAGGAGTT
				Outer Reverse	AAACCCCCCRTATCCCA
				Inner Forward	TGGGGAAGGGTGAGGTTTGT
				Inner Reverse	CCCACTACAACTCCRACC
10	*SFMBT2*	Major promoter	-	Outer Forward	GGATAGTTAGTTTTTTAATAAG
				Outer Reverse	ACCTATAAATTAATATACAAACCTA
		LTR	rs719809	Outer Forward	ATTTAAGTAAGAAGTGTTAG
				Outer Reverse	TATAAAAAACTCTCCTCCTT
				Inner Forward	TATAGAGTGGTTAGTTTAAAT
				Inner Reverse	ATTTAAACTAACCACTCTATA
12	*SLC38A1*	-	-	Outer Forward	GAGGGGTAGAGTATTAGGAAGG
				Outer Reverse	ATATATTTTCCTATAACTATAACA
				Inner Forward	TAAATATGTTTCGGTTTAGTGG
				Inner Reverse	ACCAAATCTAATTCCATTTTTA
12	*SLC38A4*	Upstream 5' UTR/ CpG island	rs74851348	Outer Forward	GTTGGATGTGGGTTTTTGGTTTTTG
				Outer Reverse	TCTCACTTTCTTCCTTCATT

				Inner Forward	GTTGGAGTGAAGGGTAGGG
				Inner Reverse	CCATCAACTCTAACCTATAATCA
		Promoter	-	Outer Forward	GTTGTTTTGTTTGTAATGTTGG
				Outer Reverse	ATCTTTTTCTCTTTCCCCTTCCA
				Inner Forward	GGTTGGGGGTTTTTAGTG
				Inner Reverse	CCTAAACCCTCTAACCAAATTACCA
12	*FAM101A*	NM_001204299	rs12823740	Outer Forward	GTATTTGTGAGGTGTTTTTGAGG
				Outer Reverse	ACTCCAACAACAATAAAACCTAA
				Inner Forward	GGGTTAGGGAGYGGGTTGGG
				Inner Reverse	TACCCRCTACCCAACCCT
		NM_001365156	-	Outer Forward	TGGTTGTTATTGGTTATTTT
				Outer Reverse	AAAAAACTTACTAAACAACCCC
				Inner Forward	GGGTAGGTAGAGGAGGAGGTA
				Inner Reverse	CCCGCCCTTCTACTCCCTAAC
14	*SMOC1*	-	rs146095118	Outer Forward	GTTTAGGYGTTTAATTTGTTG
				Outer Reverse	ACAACTACACCAACACCAACAA
				Inner Forward	GTTTATGATTGTGTTTTTTG

				Inner Reverse	AACATAATACCAACCAAACTA
		Line	rs57856791	Outer Forward	TTAGGTTTATTGTTTGGATT
				Outer Reverse	AAATTCCTTTAAACTCAACTC
				Inner Forward	AGATATTTTGGAGAGTATTTTGA
				Inner Reverse	AATAAATTCTAAATCCCAAATC
15	*SNURF*	-	rs4906939	Forward	GTTGTTGTATTAGTTAGGTGAAGG
				Reverse	AATAATATATATTCAACTTCTACTA
16	*SALL1*	-	rs11643654	Outer Forward	ATATTAGGGGTAAAGGGA
				Outer Reverse	ATACCACTCRAAATACCCA
				Inner Forward	TATTGTGTTTTAGTTTTAT
				Inner Reverse	CTAAACCCCRACAAAACTC
17	*LOC339166*	-	rs12450161, rs12450165, rs12453225	Forward	GTTAAAAGGTAATTTGTAATTTGAGG
				Outer Reverse	ACTTCTCTAAACCAACCTCTCTAAAACT AA
				Inner Reverse	TAACTAAATTTAAAAAACCTAC
17	*PLXDC1*	-	rs188501857	Forward	GAGGTYGTAGTTTTTAGTTT
				Outer Reverse	AATCCTACCTACTCCRAACTAAAA
				Inner Reverse	AAACACCAACACCAAAAACCAA
18	*ZFP516*	-	-	Forward	TAAGGTTTAAGGTTGTTGTAGTTT
				Outer Reverse	CTTAATATATTAATCCTACATC
				Inner Reverse	CTAAACACCCCCAAAAACATTTACC

19	*MBD3*	-	rs8104174	Outer Forward	GAGGGGATYGTAGGATTGGGTTTT
				Outer Reverse	ACCACCCCAACAACAAAATCAAA
				Inner Forward	TGTGATTATAGTTTATTGTAGT
				Inner Reverse	CACAACRACCCCAACCTTCCC
				Unmethylated Inner Reverse	CAACAACCCCAACCTTCCCAACCA
19	*NUDT19*	-	rs8108621, rs8109823, rs61732600	Forward	GYGGGAGGTTTTGAGGAGG
				Reverse	CAACAAAACCCTAAACAAAC
20	*ZFP64*	-	-	Outer Forward	TGTAAAGTAAGTTGTATTT
				Outer Reverse	AAATCTCCCCRCAAACCACCC
				Inner Forward	TTTTAATTTGGTTTTGTAGT
				Inner Reverse	CCTAAAATTACAAATACAAAAAAC
		mDMR	-	Forward	GGGTTTTTTTAAGAGATTGG
				Reverse	ACCTTTACCGTCCACAATAAACA
X	*XIST*	-	rs41305409	Outer Forward	GGTTAGTATGGTGGTGGATATGT
				Outer Reverse	AAATTATACAACAATCCAACACTATCC
				Inner Forward	GTAGGGATAATATGGTAG
				Inner Reverse	CACTATCCATCCCACCTTTTC
**Allelic RT-PCR**					
4	*JADE1*	NM_001287441, NM_024900	rs13114904	Forward	AGGCTGGAGTGCAGTGGCGTGA
				Reverse	CAAATAACTGCAACTCTCTGGGC

		NM_001287437	rs11933240	Forward	AGGAGAAGCATCTTGGCTTCTTGA
				Reverse	CAAATAACACAAACTTCTCAC
		New embryos	rs11933240	Forward	TGATGCGGAAGCCCTTTGGGCT
				Reverse	CAAATAACACAAACTTCTCAC
4	*GAB1*	All isoforms	rs1397529	Forward	AGATGAATTGTAGACTAGTAACA
				Reverse	GATAGTTTAGGCACATTTCAGG
		AK295684 nested RT-PCR (Out/Out)	rs62337524	Forward	TTAGAAGCCTGCCCCAGAGTCT
				Outer Reverse	TGGATCTCCAGTTAAACGGCCACT
		AK295684 nested RT-PCR (Out/In)		Forward	TTAGAAGCCTGCCCCAGAGTCT
				Inner Reverse	CTTCTCTGTACCTCTGACTTC
		New embryos	rs1269389517, rs1360288278	Forward	CCAGGAACATTTGATTTTTCC
				Reverse	AAAGTCACCTATGGTTTGTGA
		New embryos	rs28924077	Forward	AAAGGACCTTTCTGACATAATC
				Reverse	TTGGGAAAACCCAGAACAATG
7	*PRKAG2*	NM_024429 nested RT - PCR (Out/Out)	rs8961	Outer Forward	TCATGCTGATCGCTGTCCTCCTCCT
				Reverse	CAACATCACTGGAAGAAATAC
		NM_024429 nested RT - PCR (In/Out)		Inner Forward	ACAAAAGGAGACAGAAACGGA
				Reverse	CAACATCACTGGAAGAAATAC
10	*SFMBT2*	Not nested	rs10795530	Inner Forward	AGGTTGTAATGCAGTGGCGCA
				Outer Reverse	CAGATGTTCTAGGCTTCAATC
		Nested RT – PCR (Out/Out)		Outer Forward	CTTGGCCAAGATATTTCAGGAGC
				Outer Reverse	GTGGCTCACACCTGTAATCCCAG
		Nested RT -PCR (In/Out)		Inner Forward	AGGTTGTAATGCAGTGGCGCA
				Inner Reverse	GTGGCTCACACCTGTAATCCCAG
10	*GSTO1*	-	rs4925	Forward	TCCTTGGTAGGAAGCTTTAT

				Reverse	CATAGAGATAGAATTGCCAC
12	*SLC38A1*	Placenta samples	rs1045278	Forward	GGAGATAAAGGAACTCAAAG
				Reverse	ATATGATTGTATGAAATTTGAAAAA
		New embryos	rs1045278	Forward	CATGTCCCTCCAAGATTTGAGATC
				Reverse	TGTATAATAAATAAACATTATTGT
		New embryos	rs3498, rs61923106	Forward	AGGAGGAGGGTGAAGGAGGGTGA
				Reverse	CTATGCAGCAGCATCCTTTTC
		New embryos	rs1938843414	Forward	CAAGTAAGGAATATTTAGAC
				Reverse	TTCTTCTCCCCAGCTTCTGT
12	*SLC38A4*	-	rs2429467	Forward	CCTCGGGACACCCCACTCACAC
				Reverse	TGCTCATTGCTGCCTTTTCT
12	*FAM101A*	All isoforms	rs12318072	Forward	GAGCATCAAGGTGAACCCGG
				Reverse	TCCGGAAAGTGCTCCTGGCAT
		NM_001365156 nested RT – PCR (Out/Out)		Outer Forward 2	AGACATGGTGGGCCACCTGCA
				Outer Reverse 2	CTTGGGGAAGATGATGGTGGTGC
		NM_001365156 nested RT – PCR (In/Out)		Inner Forward 2	TCTACTCCCTGGCGCCCGGCAT
				Outer Reverse 2	CTTGGGGAAGATGATGGTGGTGC
		NM_181709 nested RT – PCR (Out/Out)		Outer Forward 3	GAAGCCTCTCAGCCGTAGGCG
				Outer Reverse 3	TCCGGAAAGTGCTCCTGGCAT
		NM_181709 nested RT – PCR (In/In)		Inner Forward 3	TGCAACTCTGAGGTCAAGT
				Inner Reverse 3	CTTGGGGAAGATGATGGTGGTGC
13	*STARD13*	AK308453 nested RT – PCR (Out/Out)	rs5011113	Outer Forward	GGATCTGCTGTGGAAGAACG
				Reverse	ATCCTTGGGAATATGCACCA
		AK308453 nested RT – PCR (In/Out)		Inner Forward	CTCCAGAATTCGCCGCCACC
				Reverse	ATCCTTGGGAATATGCACCA

14	*SMOC1*	-	rs3742909	Forward	ACTTGCTGCTGGTGTTGGTGCA
				Reverse	CATCTACCTCGATGCACCACGC
16	*SALL1*	-	rs11645288	Forward	TGCCACATCCCCAGTTCTGCT
				Reverse	CATGGGGCCATCCACAGAGAGC
17	*LOC339166*	-	rs12453225	Forward	GCTGGATTTGAGGAGCCTGCATG
				Reverse	AGGGAGATGGCCAAAACACTGA
18	*ZNF516*	Placenta samples	rs690353	Forward	GTCCAGGGGCGACGCGGCCTTG
				Reverse	TGTGCTCCAACCCAGGGCCGCT
		New embryos	rs72973711	Forward	TGTTAGGAATGTCAGGGACT
				Reverse	CAGCTCCAAAGGCCAACTGCAC
		New embryos	rs2074488845	Forward	GGAAGGACGGCATTCACATAG
				Reverse	TGGCCTGTGGTTGTTTCATCTGTTT
19	*MBD3*	AK001474 nested RT – PCR (Out/Out)	rs190802753	Outer Forward	GAGGCCTGGGTTTGGGGTCTG
				Reverse	GCCAGGAGCACGGCCTTCCTCCTG
		AK001474 nested RT – PCR (In/Out)		Inner Forward	AGGAGGAGGAGCCCGACCCGGA
				Reverse	GCCAGGAGCACGGCCTTCCTCCTG
		All isoforms		Forward 2	AGGAGGAGGAGCCCGACCCGGA
				Reverse	GCCAGGAGCACGGCCTTCCTCCTG
		All isoforms		Forward 3	AAAGCCTTCATGGTGACCGA
				Reverse	GCCAGGAGCACGGCCTTCCTCCTG
19	*NUDT19*	-	rs8108621, rs8109823, rs61732600	Forward	GCGCCGTGCGGGAGGCCTTTG
				Reverse	TCAGTTGCCTCTGATGGAGAT
20	*ZFP64*	-	rs3746413	Forward	CGATCCCACACGGGGGACGCC
				Reverse	TCTAGAGCCTCAGTCTTAACCAT
**qRT-PCR SYBR™ Green**					
4	*GAB1*	AK295684 - test gene	-	Forward	GAAGTCAGAGGTACAGAGAAG
				Reverse	CGGCCACTGCGTAACACGAACC
		NM_002039, NM_207123 - test gene		Forward 2	GAAAAAGTTGAAGCGTTATGCATGG
				Reverse	CGGCCACTGCGTAACACGAACC
				Forward 5	GAACCCAAACCTGTCCAGTGAAG

		All isoforms; test gene		Reverse 3	ATCATAGGGCTGCTTCCTCCATCA
7	*ACTB*	Endogenous control	-	Forward	CCGGCTTCGCGGGCGACGAT
				Reverse	CTCCATGTCGTCCCAGTTGG
7	*PEG10*	Canonical imprinting - control gene	-	Forward	GGACCCCATCCTTCCTGT
				Reverse	TTCAAAACCCGCTTATTTCG
10	*SFMBT2*	Test gene	-	Forward	CAGCAGAGGAAGGGGAGAAGTGC
				Reverse	ACGTCGGTGACCGTCCACTCCA
12	*SLC38A1*	Test gene	-	Forward	CAAATTCCCTGCATTGTTCCAGAGC
				Reverse	TGGCAAACAAATGCAAATGCAATGGT
12	*SLC38A4*	Test gene	-	Forward	TCATGGTTCGCCTGGCAGT
				Reverse	GCAATAAGCACAGCTGCAATCAG
17	*RPL19*	Endogenous control	-	Forward	AATCGCCAATGCCAACTCCCGTCA
				Reverse	CCTATGCCCATGTGCCTGCCCTTC
19	*DNMT1*	Placenta imprint - control gene	-	Forward	ACCAAGAACGGCATCCTGTA
				Reverse	CACGGGACTGGACAGCTT
20	*ZFP64*	Test gene	-	Forward	CGATCCCACACGGGGGACGCC
				Reverse	AGCGGACATTGCAGAACTCGCA
**Mouse RT - PCR**					
10	*Mbd3*	3' UTR	rs239651917, rs3401378677, rs260488938, rs212289214, rs237566955, rs582356198, rs264173787	Forward	AGAAGAACTGGTCAGGACCATGGA
				Reverse	CTGAAGGCAGTCTGCAGCCCAGAC
		5' UTR	rs239670010, rs252459742, rs253068355, rs217960000	Forward	ACGCCTGCGCAGACGAGCCCCA
				Reverse	CATAGCGCACACGCTGGCGACTC
**Mouse Bisulphite PCR**					

10	*Mbd3*	-	rs585492322, rs233179056, rs250957823, rs8256341	Forward	GTTTAGTTAGAGTTTGAATGGTG
				Inner Reverse	TCCATAAACCTCAACACCTT
				Outer Reverse	CAACAAACCACAACTAACAC
**Cloning**					
pGEM®-T Easy Vector		-	-	Outer Forward	GATGGTGCTGCAAGGCGATTAAGTTG
				Outer Reverse	ATGTTGTGTGGAATTGTGAGCGGA
				Sp6	ATTTAGGTGACACTATAG
				MF13 Forward	GTAAAACGACGGCCAG
				Seq(S) T7	TAATACGACTCACTATAGGG
